# Characterization of cross-species transcription and splicing from *Penicillium* to *Saccharomyces cerevisiae*

**DOI:** 10.1093/jimb/kuab054

**Published:** 2021-08-13

**Authors:** Zhenquan Lin, Kang Xu, Guang Cai, Yangqingxue Liu, Yi Li, Zhihao Zhang, Jens Nielsen, Shuobo Shi, Zihe Liu

**Affiliations:** College of Life Science and Technology, Beijing Advanced Innovation Center for Soft Matter Science and Engineering, Beijing University of Chemical Technology, 100029 Beijing, China; College of Life Science and Technology, Beijing Advanced Innovation Center for Soft Matter Science and Engineering, Beijing University of Chemical Technology, 100029 Beijing, China; College of Life Science and Technology, Beijing Advanced Innovation Center for Soft Matter Science and Engineering, Beijing University of Chemical Technology, 100029 Beijing, China; College of Life Science and Technology, Beijing Advanced Innovation Center for Soft Matter Science and Engineering, Beijing University of Chemical Technology, 100029 Beijing, China; College of Life Science and Technology, Beijing Advanced Innovation Center for Soft Matter Science and Engineering, Beijing University of Chemical Technology, 100029 Beijing, China; College of Life Science and Technology, Beijing Advanced Innovation Center for Soft Matter Science and Engineering, Beijing University of Chemical Technology, 100029 Beijing, China; College of Life Science and Technology, Beijing Advanced Innovation Center for Soft Matter Science and Engineering, Beijing University of Chemical Technology, 100029 Beijing, China; Department of Biology and Biological Engineering, Chalmers University of Technology, SE-412 96 Gothenburg, Sweden; BioInnovation Institute, Ole Maaløes Vej 3, DK 2200 Copenhagen N, Denmark; College of Life Science and Technology, Beijing Advanced Innovation Center for Soft Matter Science and Engineering, Beijing University of Chemical Technology, 100029 Beijing, China; College of Life Science and Technology, Beijing Advanced Innovation Center for Soft Matter Science and Engineering, Beijing University of Chemical Technology, 100029 Beijing, China

**Keywords:** Heterologous expression, Splicing, *Penicillium* species, Cross-species recognition, *Saccharomyces cerevisiae*

## Abstract

Heterologous expression of eukaryotic gene clusters in yeast has been widely used for producing high-value chemicals and bioactive secondary metabolites. However, eukaryotic transcription *cis*-elements are still undercharacterized, and the cross-species expression mechanism remains poorly understood. Here we used the whole expression unit (including original promoter, terminator, and open reading frame with introns) of orotidine 5′-monophosphate decarboxylases from 14 *Penicillium* species as a showcase, and analyzed their cross-species expression in *Saccharomyces cerevisiae*. We found that *pyrG* promoters from the *Penicillium* species could drive *URA3* expression in yeast, and that inefficient cross-species splicing of *Penicillium* introns might result in weak cross-species expression. Thus, this study demonstrates cross-species expression from *Penicillium* to yeast, and sheds light on the opportunities and challenges of cross-species expression of fungi expression units and gene clusters in yeast without refactoring for novel natural product discovery.

## Introduction

Natural products produced by eukaryotic systems have served as a crucial source of pharmaceuticals and nutraceuticals (Bérdy, 2005; Nielsen, 2019). For instance, many natural fungal products have been developed as essential drugs and life-enhancing medicines, such as the broad-spectrum antibiotics penicillin from *Penicillium* and cephalosporin from *Acremonium* (Ashtekar et al., 2021), the cholesterol-lowering drug lovastatin from *Aspergillus terreus* (Tobert, 2003), and the antifungal compound griseofulvin from *Penicillium griseofulvum* [Pg] (Petersen et al., 2014). However, compared with prokaryotic natural products, identification and heterologous expression of eukaryotic natural products have attracted less attention (Nielsen et al., 2017).

Natural product biosynthesis pathways are often organized in clusters in the genome, known as biosynthetic gene clusters (BGCs) (Kunakom & Eustáquio, 2019). Around 99% of environmental microbes with the potential to produce novel natural products are unculturable under laboratory conditions. Thus, heterogeneous expression of putative BGCs in well-characterized microbial cell factories has attracted recent attention (Cook & Pfleger, 2019; Xu et al., 2020). However, since eukaryotic genes have introns and require individual promoters and terminators for transcription and posttranscription modifications, and many eukaryotic BGCs have more than 10 genes, it is time consuming and challenging to clone and refactor target eukaryotic BGCs, not to mention multiplexing. Moreover, many BGCs are not expressed under normal conditions or derived from unculturable samples, making it impossible to obtain intron-free cDNAs. Therefore, it is crucial to understand cross-species transcription and splicing mechanisms and develop methods that allow heterologous expression of eukaryotic expression units (including original promoter, terminator, and open reading frame [ORF] with introns) and even eukaryotic BGCs from distant species without refactoring and removal of intron sequences.

*Saccharomyces cerevisiae* [Sc], as a model eukaryote and widely applied microbial cell factory, has been developed for efficient expression of various metabolic pathways from distant hosts (Alberti et al., 2017). Heterologous promoters and terminators from other yeasts, such as *Ashbya gossypii, Saccharomyces kudriavzevii* and *Saccharomyces bayanus*, can be recognized by the native transcription factor of *S. cerevisiae*, and have been used to develop orthogonal genetic systems in *S. cerevisiae* to prevent cross talks (Harvey et al., 2018; McCusker, 2017). Anthony DeNicola engineered the *S. cerevisiae* spliceosome and enhanced the splicing efficiency of introns from *Aspergillus fumigatus* (DeNicola, 2018). Thus, it is important to characterize the efficiency and mechanism of cross-species expression of expression units in *S. cerevisiae* for achieving high-throughput library expression of heterologous BGCs without refactoring (Liu et al., 2021).

The genus *Penicillium* includes more than 354 species, and is widely known for producing diversified ranges of important bioactive natural products (Visagie et al., 2014). Here, we use *Penicillium* and the model yeast *S. cerevisiae* as a showcase to study cross-species transcription and splicing in eukaryotic systems. Briefly, whole expression units encoding orotidine 5′-monophosphate decarboxylase (EC 4.1.1.23) from 14 *Penicillium* species were functionally characterized in *S. cerevisiae*. We demonstrated that many *Penicillium* transcription *cis*-elements could be recognized by native transcription factor and the spliceosome of *S. cerevisiae*, and we found that the GC content in the intron sequence may be important for further engineering.

## Material and Methods

### Strains and Media

*Escherichia coli* strain DH5α (lab collection) was used for plasmid construction. *S. cerevisiae* BY4741 (*MATa his3Δ1 leu2Δ0 met15Δ0 ura3Δ0*) and 14 *Penicillium* species (*Penicillium coprophilum* [Pc] CGMCC No. 3.13605, *Penicillium nalgiovense* [Pn] CGMCC No. 3.4357, *Penicillium polonicum* [Ppo] CGMCC No. 3.13618, *Penicillium vulpinum* [Pv] CGMCC No. 3.8070, *Penicillium solitum* [Pso] CGMCC No. 3.7896, *Penicillium decumbens* [Pd] CGMCC No. 3.7962, *P. griseofulvum* CGMCC No. 3.11290, *Penicillium brasilianum* [Pb] CGMCC No. 3.4402, *Penicillium digitatum* [Pdp] CGMCC No. 3.13921, *Penicillium expansum* [Pe] CGMCC No. 3.7898, *Penicillium italicum* [Pi] CGMCC No. 3.7899, *Penicillium chrysogenum* [Pcs] CGMCC No. 3.15509, *Penicillium roqueforti* [Pro] CGMCC No. 3.7903, *Penicillium paneum* [Ppa] CGMCC No. 3.628) obtained from China General Microbiological Collection Center (CGMCC) were used for characterizing the cross-species expression. *S. cerevisiae* was grown in yeast extract–peptone–dextrose (YPD, peptone 20 g/l, yeast extract 10 g/l, and glucose 20 g/l) medium for preparation of component cells, and transformants were selected on synthetic complete (SC) media (composed of (NH_4_)_2_SO_4_ 5 g/l, yeast nitrogen base without amino acids 1.7 g/l, amino acid mixture without uracil 1.914 g/l, and glucose 20 g/l) without auxotrophic compounds that complemented by the plasmids.

### Genomic DNA Isolation

*Penicillium* strains were cultured on malt extract agar for 5 days at 26°C to induce sporulation. Conidiophores were eluted with sterile water and inoculated in a malt extract liquid medium for 3 days at 25°C, 160 rpm. The details of genomic DNA extraction has been previously described (Grijseels et al., 2016).

### Plasmid Construction

Circular polymerase extension cloning (CPEC) (Quan & Tian, 2011) was employed to clone and refactor *pyrG* transcription units (Supplementary Fig. S1). Plasmid pRS415 was used as the backbone. DNA concentrations were measured using a Nanodrop 2000c spectrophotometer (Thermo Scientific, Wilmington, DE), and CPEC reactions were carried out using 100 ng backbone vector and equimolar inserts in a 25 μl reaction system (containing 1 Unit Q5^®^ polymerase, 0.2 mM dNTPs, 1 × Q5^®^ reaction buffer, 3% DMSO) (New England BioLabs, Beijing). The reaction product was transformed into *E. coli* and screened on LB agar plates with 100 μg/ml ampicillin overnight at 37°C. Primers used in this study are listed in Supplementary Table S1.

To insert the flag-tag into the ORF flanking the start codon ATG, 40 bp overlapping sequences with the flag-tag sequence were introduced at the 5ʹ ends of the primers used in the amplification reactions. Plasmids *ScTDH3p-ScURA3-ScADH1t, pyrGp-ScURA3-ScADH1t, ScTDH3p-pyrG-ScADH1t, pyrGp-pyrG-ScADH1t*, and *pyrGp-pyrG-pyrGt* derived from different *Penicillium* species were amplified into two pieces and then reassembled respectively using CPEC. A similar procedure was used to replace *Penicillium pyrG* intron sequences with either *MATa1* intron from *S. cerevisiae* or insert intron sequences from *Penicillium pyrG* genes into the *S. cerevisiae URA3* under the control of yeast *ScTDH3* promoter. For removal of the intron sequences from *Penicillium pyrG* genes under the control of *ScTDH3* promoter, 40 bp overlapping sequences with the cross-intron sequence were introduced at the 5ʹ end of the primers used in the PCR amplification reactions. All yeast strains were transformed using the LiAc/SS carried method as reported previously (Gietz & Schiestl, 2007).

### Growth Assay

A single colony was inoculated in a 5 ml synthetic complete medium without L-leucine (SC-LEU) at 200 rpm, 30°C overnight. Yeast cells were collected in 50 ml falcon tubes by centrifugation at 3,000 rpm for 3 min, and washed twice with 5 ml of sterilized water. Cells were then resuspended using SC-LEU-URA medium, inoculated into 50 ml of SC-LEU-URA medium with initial OD600 at 0.1, and cultured at 30°C at 220 rpm. Cells were normalized by OD and serially diluted into sterile water for serial dilution assays on SC-LEU-URA plates. Serial dilution plates were incubated at 30°C for 3–14 days before imaging.

### Cell Lysis

Yeasts were cultured in SC-LEU medium and harvested into cell pellet when OD600 reached 1. The pellet was then liaised using 200 μl of 0.4 M NaOH, centrifuged at 7,000 rpm for 5 min and resuspended in 100 μl of 2 × loading buffer (containing 4% SDS, 20% glycerol, 120 mm Tris–HCl pH 6.8, 5% (vol/vol) 2-mercaptoethanol). Samples were denatured at 90°C for 5–10 min.

### Sodium Dodecyl Sulphate–Polyacrylamide Electrophoresis and Western Blotting Assay

Denatured samples were loaded on a 12% acrylamide sodium dodecyl sulphate–polyacrylamide electrophoresis (SDS-PAGE) gel, prepared as previously reported (Sattlegger et al., 2013). After gel electrophoresis in 1 × Tris–glycine buffer, proteins were transferred to a 0.22 μm PVDF via the semi-dry transfer method at 20 V for 20 min (Trans-Blot^®^ SD Semi-Dry Transfer Cell, Bio-rad), using 1 × Tris glycine buffer containing 20% methanol.

Western blotting assay was performed as previously described (Sattlegger et al., 2013). Primary antibodies were directed against the FLAG tag (FLAG-tag rabbit polyclonal antibody, 1:5,000, Huaxingbio, No. HX1819). The secondary antibody containing horseradish peroxidase conjugated to Goat anti-Rabbit antibodies (Huaxingbio, No. HX2031) was incubated with a dilution of 1:5,000. The horseradish peroxidase was visualized using an ECL chemiluminescence detection reagent (Super ECL, No. HXP1868, Huaxingbio) as described by the manufacturers, and imaged with a C300 imager (Azure Biosystems, Inc., Dublin).

### Real-Time Polymerase Chain Reaction

Recombinant strains were cultured in SC-LEU medium and harvested when OD600 approached 1. Total RNA was extracted using TRIzol^®^ reagent (Invitrogen, USA) as described by the manufacturer and RNA concentration determined by Nanodrop 2000c. In total, 1 μg RNA was treated with gDNA Eraser (TAKARA, Dalian) according to manufacturer's instructions. The first-strand cDNA was amplified using PrimeScript^™^ RT reagent Kit (TAKARA, Dalian). Real-time polymerase chain reaction (RT-PCR) was performed using a QuantStudio 3 system (Applied Biosystems, USA) with TB Green^®^ Premix Ex Taq^™^ II (TAKARA, Dalian). Primers used for RT-PCR are listed in Supplementary Table S1. The *ACT1* gene was selected as the reference gene for normalization, and the 2^−^^ΔΔCt^ method was used to analyze the results (Livak & Schmittgen, 2001).

## Results

### Bioinformatics Characterization of Orotidine 5′-Monophosphate Decarboxylases from fourteen *Penicillium* Species

Orotidine 5′-monophosphate decarboxylase, encoded by *URA3* in yeast and *pyrG* in *Penicillium*, catalyzes the synthesis of uridine 5′-monophosphate in the pyrimidine biosynthetic pathway (Siewers, 2014). Here, we used orotidine 5′-monophosphate decarboxylase as a showcase to characterize the cross-species transcription and splicing ability of *S. cerevisiae* to express whole expression units from *Penicillium*. To determine sequences of *Penicillium pyrG*, we performed TBLASTN searches in the NCBI database using the amino acid sequence of *S. cerevisiae URA3* (Alani & Kleckner, 1987) and the previously identified *P. chrysogenum pyrG* gene (Fierro et al., 1993). Amino acid sequence alignment was analyzed by the Bioedit ClustalW program (Hall, 1999). The identified DNA sequences of *pyrG* genes from *P. coprophilum, P. nalgiovense, P. polonicum, P. vulpinum, P. solitum, P. decumbens, P. griseofulvum, P. digitatum, P. expansum, P. italicum, P. chrysogenum, P. brasilianum, P. roqueforti*, and *P. paneum* are shown in Supplementary Table S2. Compared against *S. cerevisiae* Ura3p, the amino acid sequences of orotidine 5′-monophosphate decarboxylase is highly conserved among the 14 selected *Penicillium* species, with identity scores all over 90% (Supplementary Fig. S2).

### Cross-Species Expression of *pyrG* ORFs from *Penicillium* to Yeast

**Fig. 1:**
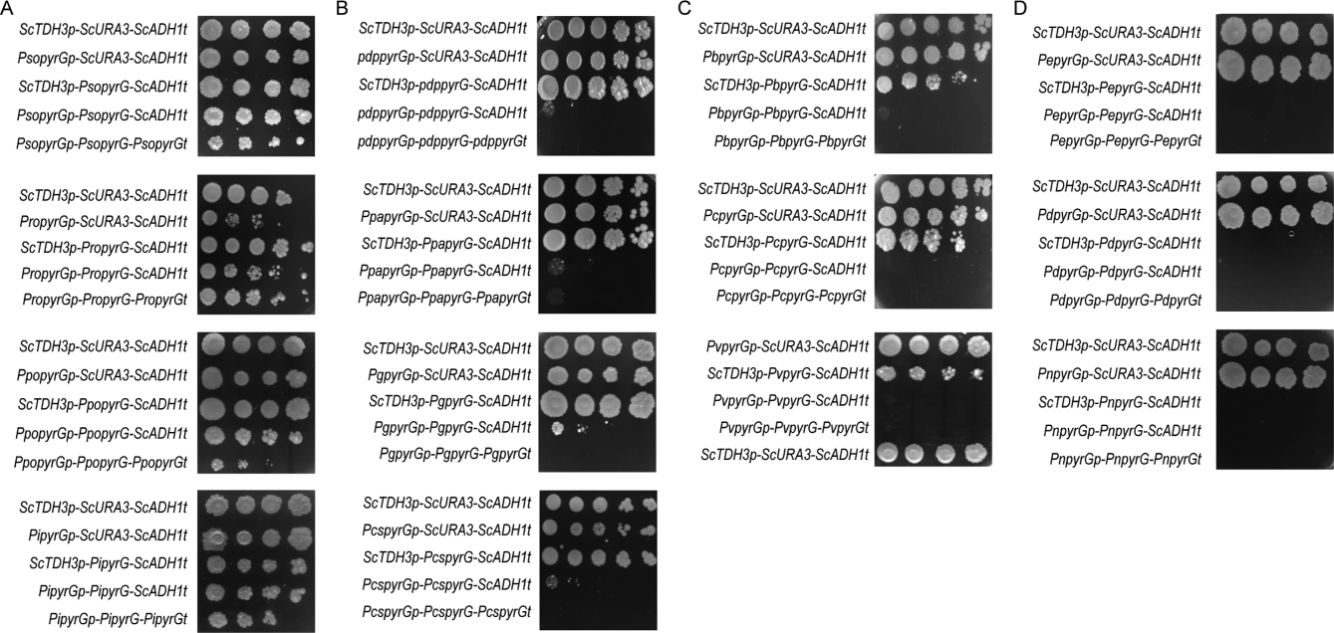
Characterization of cross-species unit-expression of orotidine 5′-monophosphate decarboxylases from *Penicillium* to yeast. In total, 52 recombinants derived from 14 *Penicillium* species were constructed. For each *pyrG* expression unit, four constructs were tested and compared with the control stain harboring the native expression unit of yeast *URA3* (*ScTDH3-ScURA3-ScADH1t*), including the expression unit that only replace the yeast promoter with the *Penicillium* promoter (*pyrGp-ScURA3-ScADH1t*), the expression unit that only replace yeast *URA3* with *Penicillium pyrG* (*ScTDH3-pyrG-ScADH1t*), the expression unit that replaces both yeast promoter and yeast *URA3* with *Penicillium*’s (*pyrGp-pyrG-ScADH1t*), and the construct that replaces the whole yeast *URA3* expression unit *Penicillium*’s (*pyrGp-pyrG-pyrGt*). Pso, *Penicillium solitum*; Pro, *Penicillium roqueforti*; Ppo, *Penicillium polonicum*; Pi, *Penicillium italicum*; Pdp, *Penicillium digitatum*; ppa, *Penicillium paneum*; Pg, *Penicillium griseofulvum*; Pcs, *Penicillium chrysogenum*; Pb, *Penicillium brasilianum*; Pc, *Penicillium coprophilum*; Pv, *Penicillium vulpinum*; Pe, *Penicillium expansum*; Pd, *Penicillium decumbens*; Pn, *Penicillium nalgiovense*.

**Fig. 2:**
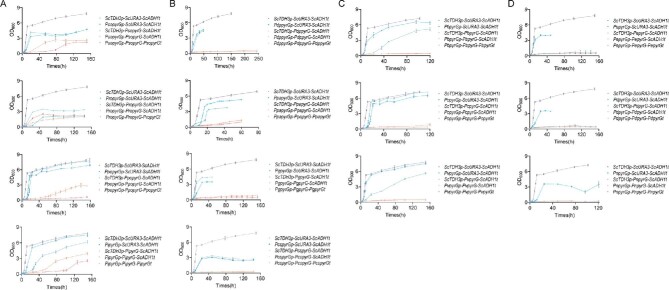
Growth profiles of cross-species unit-expression of orotidine 5′-monophosphate decarboxylases from *Penicillium* to yeast. Error bars represent standard deviations of three biological replicates.

First, cross-species expression of *Penicillium pyrG* ORFs was evaluated in the *URA3*-deficient *S. cerevisiae* BY4741. Fourteen *pyrG* ORFs from different *Penicillium* species were individually cloned into the shuttle vector pRS415 under the control of the *S. cerevisiae TDH3* promoter and *ADH1* terminator to obtain the expression unit *ScTDH3p-pyrG-ScADH1t*. As an additional control, all recombinant strains were compared in parallel to the strain that expressed yeast *URA3* in the same construct, *ScTDH3p*-*URA3*-*ScADH1t*. As shown in Fig. 1, cross-species expression of *Penicillium pyrG* ORFs with introns grew well on the SC-LEU-URA agar plate except for the *pyrG* from *P. expansum, P. decumbens*, and *P. nalgiovense*. In order to detect changes in strain fitness and provide more evidence, the complementation experiment was also performed in liquid cultures (Fig. 2). All 11 *pyrG* strains growing in spotting assays displayed longer lag phases and lower final OD than the *URA3* strain (Fig. 2A, B, C). Most evaluated *pyrG* strains reached more than 50% of the final OD obtained with the *URA3* strain (Fig. 2A, B, C), with the strain harboring *pyrG* from *P. polonicum* growing the best, almost reaching the same final OD of the *URA3* strain (Fig. 2A). These results revealed that although containing introns, *pyrG* genes from *Penicillium* can complement the*URA3* defect in yeast.

### Cross-Species Expression of *pyrG* Promoters from *Penicillium* to Yeast

Next, we evaluated the cross-species expression of *Penicillium pyrG* promoters in yeast. Fourteen *pyrG* promoters from different *Penicillium* species were individually cloned into the shuttle vector pRS415, together with yeast *URA3* gene and *ADH1* terminator to obtain the expression unit *pyrGp-ScURA3*-*ScADH1t*. As an additional control, all recombinant strains were compared in parallel to the strain that expressed yeast *ScTDH3* promoter in the same construct (*ScTDH3p*-*URA3*-*ScADH1t*). As shown in Fig. 1, we found that all selected *pyrG* promoters resulted in expression of the *URA3* as all strains grew well on the uracil dropout medium, suggesting that *Penicillium* promoters can be recognized as driving transcription in yeast. Although with a long lag phase (Fig. 2), 13 out of the 14 evaluated *pyrG* promoter strains reached more than 50% of the final OD compared with the yeast strain expressing the *URA3* by the *TDH3* promoter, with the strain harboring *pyrG* promoter from *P. polonicum* and *P. italicum* growing comparably to the control strain.

### Cross-Species Transcription of *pyrG* Expression Units from *Penicillium* in Yeast

To further evaluate the cross-species transcription and splicing of *Penicillium pyrG* expression units in yeast, the 14 *pyrG* promoters and ORFs from different *Penicillium* species were cloned into the shuttle vector pRS415, either with the yeast *ADH1* terminator or *Penicillium pyrG* terminator, to obtain expression units *pyrGp-pyrG-ScADH1t* and *pyrGp-pyrG-pyrGt*, respectively. The growth test suggested that the cross-species expression capacity of *Penicillium* expression units in *S. cerevisiae* exhibited four different patterns. *Penicillium* species *P. solitum, P. roqueforti, P. italicum*, and *P. polonicum* expressed the whole *Penicillium pyrG* expression units (*pyrGp-pyrG-pyrGt*), which resulted in growth on the uracil dropout plates (Fig. 1A). These results confirmed that all four yeast recombinants with whole *Penicillium* expression units (*pyrGp-pyrG-pyrGt*) also grew well in liquid culture, and the recombinant harboring the *P. italicum* expression unit grew the best, with the final OD reaching 4.6 (more than 50% of the final OD of the control strain). For *Penicillium* species *P. digitatum, P. paneum, P. griseofulvum*, and *P. chrysogenum*, only expression units with *Penicillium* promoters and *pyrG* genes (*pyrGp-pyrG-ScADH1t*) could grow, while it showed clear defects on the uracil dropout media (Fig. 1B and Fig. 2B). Regarding *Penicillium* species *P. brasilianum, P. coprophilum*, and *P. vulpinum*; although both expression units with *Penicillium* promoters (*pyrGp-URA3-ScADH1t*) and expression units with *Penicillium* ORFs (*ScTDH3p-pyrG-ScADH1t*) can be expressed in *S. cerevisiae*, the expression is so low that strains could not grow on the uracil dropout media harboring expression units with both *Penicillium* promoters with *Penicillium* ORFs (*pyrGp-pyrG-ScADH1t*) (Figs. 1C and 2C). Amongst *Penicillium* species *P. expansum, P. decumbens*, and *P. nalgiovense*, only *Penicillium* expression units with *Penicillium* promoters (*pyrGp-pyrG-ScADH1t)* could grow (Fig. 1D). Taken together, these results demonstrated that although *S. cerevisiae* has weak intron processing capacity, cross-species expression of *Penicillium* expression units in *S. cerevisiae* is possible.

### Characterization of Expression Levels of *Penicillium* Promoters and ORFs in Yeast

To determine how recombinants with expression units from different *Penicillium* species grew differently, we selected recombinants with expression units from eight *Penicillium* species to determine the degrees of transcription activity induced by *Penicillium* promoters and expression levels of *pyrG* genes in yeast. These eight *Penicillium* species include *P. solitum* and *P. roqueforti* from which the whole expression unit (*pyrGp-pyrG-pyrGt*) can express well in yeast (Figs. 1A and 2A); *Penicillium* species *P. digitatum, P. paneum, P. griseofulvum*, and *P. chrysogenum* from which only expression units with *Penicillium* promoter and ORF (*pyrGp-pyrG-ScADH1t*) could grow (Figs. 1B and 2B); *Penicillium* species *P. coprophilum* from which both *Penicillium* promoter and the ORF can be expressed in *S. cerevisiae*, whereas the expression unit with *Penicillium* promoter and ORF (*pyrGp-pyrG-ScADH1t*) could not (Figs. 1C and 2C); and *P. nalgiovense* from which only *Penicillium* promoter (*pyrGp-pyrG-ScADH1t*) could be recognized by yeast (Figs. 1D and 2D).

**Fig. 3:**
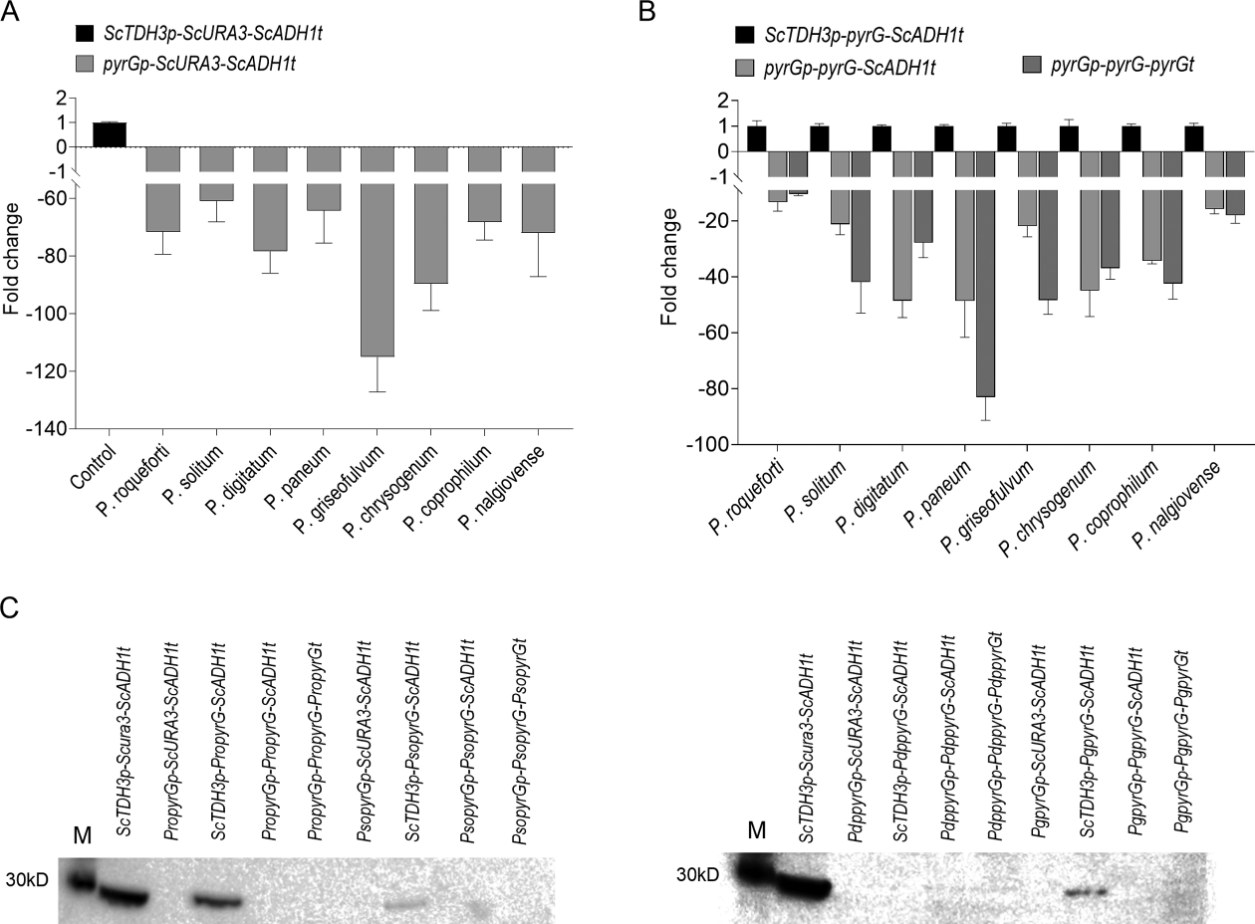
Relative expression levels of orotidine 5′-monophosphate decarboxylases in yeast. (A) Transcription levels of yeast *URA3* gene under the control of different *Penicillium pyrG* promoters and yeast *ADH1* terminator. Yeast *TDH3* promoter is used as control. (B) Transcription levels of *Penicillium pyrG* genes under controls of *Penicillium pyrG* promoters and yeast *ADH1* terminator, or *Penicillium pyrG* promoters and *pyrG* terminators. (C) Western blot analysis of *Penicillium pyrG* expression units. Pso, *Penicillium solitum*; Pro, *Penicillium roqueforti*; Pdp, *Penicillium digitatum*; Ppa, *Penicillium paneum*; Pg, *Penicillium griseofulvum*; Pcs, *Penicillium chrysogenum*; Pc, *Penicillium coprophilum*; Pn, *Penicillium nalgiovense*.

In order to determine the activities of *pyrG* promoters in yeast, quantitative RT-PCR was performed using *ACT1* as the reference gene. As shown in Fig. 3A, transcriptional levels of the reporter gene *URA3* under the control of *Penicillium pyrG* promoters were between 60- and 120-fold lower than those under the control of yeast *TDH3* promoter, with the promoter from *P. solitum* being the strongest. Using the same batch, we also tested the transcriptional level of the reporter gene *pyrG* with either *pyrG* promoters or yeast *TDH3* promoter. In Fig. 3B, the transcriptional level of the reporter gene *pyrG* under the control of *pyrGp* promoters is seen to be between 15- and 50-fold lower compared with that of yeast *TDH3* promoter, with the promoter from *P. roqueforti* being the strongest. These results suggest that for reporter genes from different origins, the relative strength of promoters was diversified. We have also noticed that when comparing the yeast *ADH1* terminator with *Penicillium pyrG* terminators from *P. roqueforti, P. chrysogenum*, and *P. digitatum*, transcription levels of *pyrG* have a minor increase (Fig. 3B). These results suggest that the 3′ flanking sequence may also influence transcript abundance, possibly affecting mRNA stability and the corresponding mRNA half-life (Matsuyama, 2019).

Western blot analysis was also performed to determine expression levels of recombinant proteins. Orotidine 5′-monophosphate decarboxylases from *Penicillium* species have 276 amino acids, with a calculated molecular weight of 29.9 kDa; while the orotidine 5′-monophosphate decarboxylase from yeast contains 265 amino acids with a molecular weight of 29.3 kDa. Recombinant *pyrG* proteins with 5ʹ flag tags were extracted during the log phase. As shown in Fig. 3C, only recombinant *pyrG* proteins derived from *P. solitum, P. roqueforti*, and *P. griseofulvum* under the control of *ScTDH3* promoter were detected, yet their expression levels were much lower than that of control strain *ScTDH3p-ScURA3-ScADH1t*. For *pyrG* proteins from *P. digitatum, P. paneum, P. griseofulvum, P. chrysogenum*, and *P. coprophilum*, where the recombinants *ScURA3p-pyrG-ADH1t* grew well on the uracil dropout medium, there were no detectable proteins during Western blot assay using the anti-flag antibody (data not shown). Similar results were also found in *pyrGp-pyrG-pyrGt* strains. When sequencing *pyrG* cDNAs derived from strains harboring expression units *ScURA3p-pyrG-ADH1t*, we noticed that entire intron sequences of most *pyrG* genes were not removed (Supplementary Fig. S3). This result indicates that yeast's weak splicing capacity needs to be improved to allow efficient cross-species expression of expression units or gene clusters.

### Evaluation of Yeast Splicing Capacity of *Penicillium* Introns

**Fig. 4:**
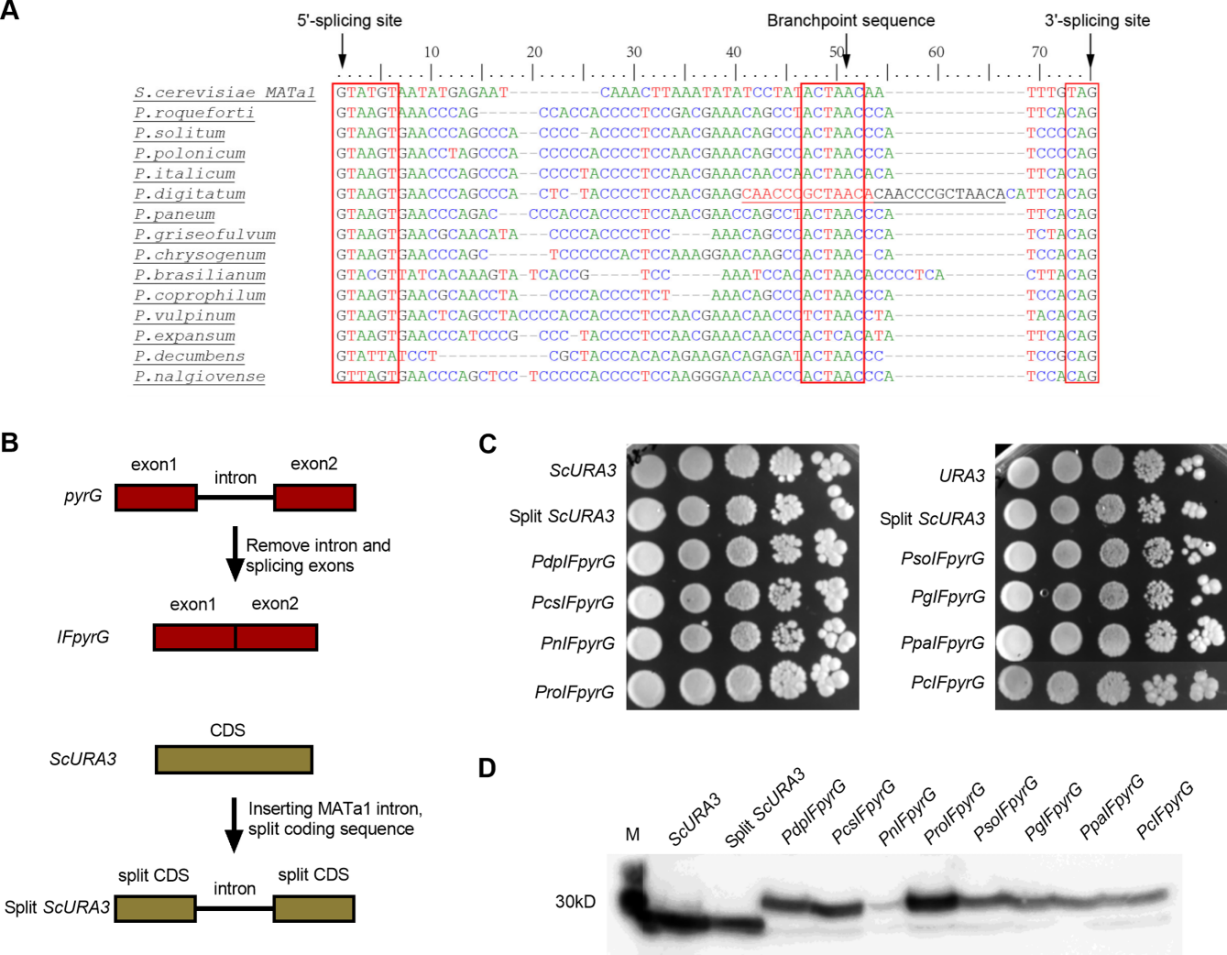
Expression of *Penicillium pyrG* genes without original introns in yeast. (A) Alignment of intron sequences of *pyrG* genes from *Penicillium* species and the second intron of *MATa1* from *Saccharomyces cerevisiae*. The alignment was performed using the BioEdit ClustalW program. Duplicate sequences are underlined. (B) Schematic of removal of intron sequences in *Penicillium pyrG* genes and yeast *URA3* inserted with the intron from *MATa1*. (C) Intron-free *pyrG* (IFpyrG) genes rescued cell growth of expression units *ScTDH3p-pyrG-ScADH1t*. (D) Western blot analysis identified expressions of all intron-free *pyrG* genes from eight *Penicillium* species. Pso, *Penicillium solitum*; Pro, *Penicillium roqueforti*; Pdp, *Penicillium digitatum*; Ppa, *Penicillium paneum*; Pg, *Penicillium griseofulvum*; Pcs, *Penicillium chrysogenum*; Pc, *Penicillium coprophilum*; Pn, *Penicillium nalgiovense*.

Analysis of the nucleic acid and the amino acid sequence of *pyrG* genes suggests that the coding sequences were interrupted by one short intron of between 36 and 72 nucleotides (Supplementary Table S2), located proximal to the 5′ ends of the ORFs. The predicted intron sequences of *pyrG* genes are shown in Supplementary Table S2. These intron sequences contain the classic three sequence elements, including the 5ʹ splicing site, the branchpoint sequence, and the 3ʹ splicing site. Consistent with the intron 5′-GT and 3′-AG of eukaryotic splicing mechanisms (Frey & Pucker, 2020; Kupfer et al., 2004), the conserved splicing signals of the 14 *pyrG* genes are the consensus sequences for the 5ʹ splicing site (5ʹ-GTAA), the 3ʹ splicing site (CAG), and the branchpoint sequence (Fig. 4A). It can also be noted that introns of *pyrG* genes from *P. nalgiovense* and *P. decumbens* have mismatches at the 5ʹ splicing site motif when compared with introns in other selected *pyrG* genes (Fig. 4A). The intron from *P. digitatum* has a 13 bp duplicate sequence between the branchpoint sequence and the 3ʹ splicing site (Fig. 4A). All *pyrG* introns display substantial sequence similarity with a high GC content between the 5′ splicing site and the branchpoint sequence.

We next evaluated splicing efficiencies of *Penicillium* introns by yeast native spliceosome. To this end, we first removed all intron sequences of *pyrG* genes from eight *Penicillium* species (Fig. 4B), including *P. roqueforti, P. solitum, P. digitatum, P. paneum, P. griseofulvum, P. chrysogenum, P. coprophilum*, and *P. nalgiovense*, and tested their expression efficiencies in the *ScTDH3p-pyrG-ScADH1t.* Since the second intron of *MATa1* (52 bp) is the shortest among the 298 annotated *S. cerevisiae* introns (Qin et al., 2016) and has been well studied in yeast (Ner & Smith, 1989; Tuo et al., 2012), *URA3* inserted with this intron was also cloned into the same construct as the controls. As shown in Fig. 4C and D, all intron-free *pyrG* (IFpyrG) genes could be detected in western blot analysis, and could support normal cell growth compared to endogenous *URA3* and *URA3* with *MATa1* introns through spotting assay. This applied even for the re-combinant with the expression unit *ScTDH3p-*IF*PnpyrG-ScADH1t* derived from *P. nalgiovense* through that with introns (*ScTDH3p-PnpyrG-ScADH1t*) could not support cell growth (Figs. 1D and 4C).

**Fig. 5:**
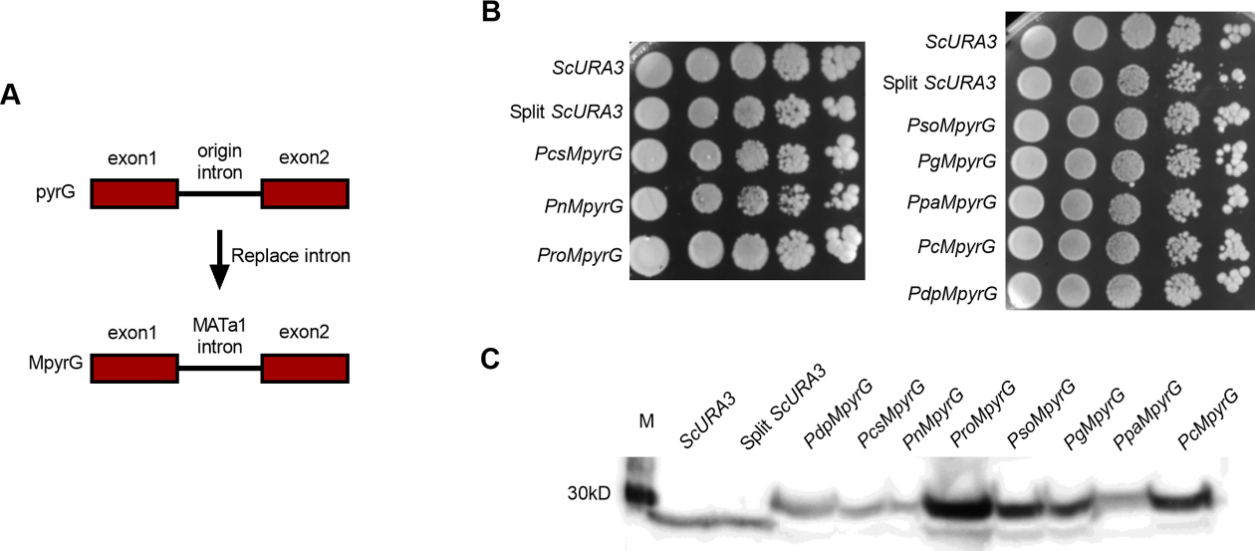
Evaluation of yeast splicing capacity of *Penicillium* genes with yeast introns. (A) Schematic of the replacement of intron sequences in *Penicillium pyrG* genes with the intron from *MATa1*. (B) Intron-replaced *pyrG* (M*pyrG*) genes rescued cell growth of *ScTDH3p-pyrG-ScADH1t* expression units. (C) Western blot analysis identified expressions of all intron-replaced *pyrG* (M*pyrG*) genes. Pso, *Penicillium solitum*; Pro, *Penicillium roqueforti*; Pdp, *Penicillium digitatum*; Ppa, *Penicillium paneum*; Pg, *Penicillium griseofulvum*; Pcs, *Penicillium chrysogenum*; Pc, *Penicillium coprophilum*; Pn, *Penicillium nalgiovense*.

Since both IFpyrG genes and *URA3* with the *MATa1* intron can be efficiently expressed in *S. cerevisiae*, we next evaluated the expression of *pyrG* genes with introns replaced by the *MATa1* intron (Fig. 5A). As shown in Fig. 5B and C, again all *pyrG* genes with introns replaced by the *MATa1* intron could be detected in Western blot analysis, and could support cell growth comparable with that of *URA3* with *MATa1* intron through spotting assay. Taken together, these results demonstrated that the predicted intron sequences from the eight selected *Penicillium* species are correct, and *Penicillium pyrG* genes without introns could complement the *URA3* defect yeast. Thus, inefficient cross-species splicing of yeast may be one of the key reasons that led to the growth defects.

**Fig. 6:**
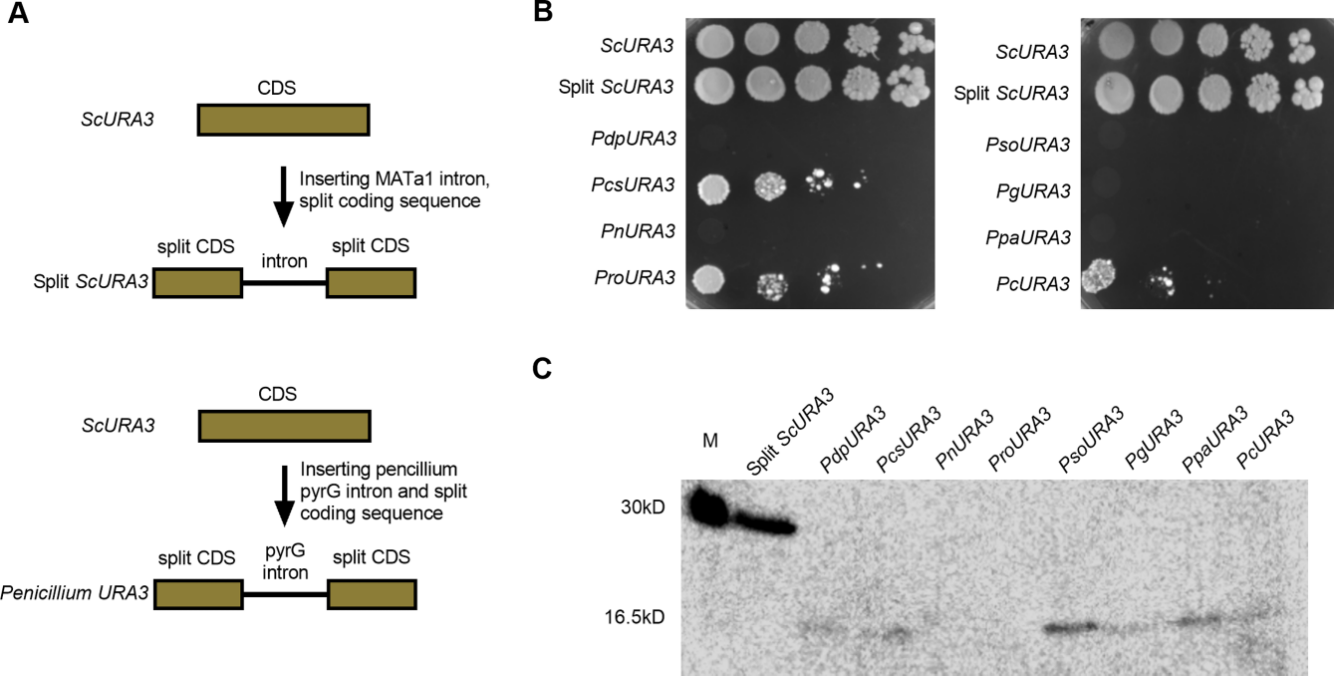
Evaluation of yeast splicing capacity of yeast *URA3* with *Penicillium* introns. (A) Schematic of yeast *URA3* inserted with *Penicillium* introns from *pyrG* genes. (B) Yeast *URA3* inserted with *Penicillium* introns reduced yeast growth compared with wild type *URA3* and *URA3* with *MAT1a* intron. (C) Western blot analysis identified truncated expressions of *URA3* inserted with *Penicillium* introns. Pso, *Penicillium solitum*; Pro, *Penicillium roqueforti*; Pdp, *Penicillium digitatum*; Ppa, *Penicillium paneum*; Pg, *Penicillium griseofulvum*; Pcs, *Penicillium chrysogenum*; Pc, *Penicillium coprophilum*; Pn, *Penicillium nalgiovense*.

To examine this hypothesis, we inserted *pyrG* introns from the eight evaluated *Penicillium* species into the yeast *URA3* gene to test whether *Penicillium* introns can be efficiently spliced in the native gene of yeast (Fig. 6A). As shown in the spotting assay in Fig. 6B, *URA3* genes with *pyrG* introns from *P. paneum, P. griseofulvum, and P. chrysogenum* could still support cell growth with significant growth defects compared with the control strains, whereas *URA3* genes with *pyrG* introns from *P. solitum, P. roqueforti, P. digitatum, P. coprophilum*, and *P. nalgiovense* could not. Western blot results showed that none of the *URA3* genes with *pyrG* introns from *Penicillium* had the correct 30 kD band, and *URA3* genes with introns from *P. solitum, P. digitatum, P. paneum, P. griseofulvum, P. chrysogenum*, and *P. coprophilum* had 16.5 kD bands instead (Fig. 6C). When analyzing sequences of these *pyrG* genes with replaced introns, we noticed that there was a stop codon within the introns, and the unspliced *Penicillium* introns would result in frame-shifted transcripts encoding the truncated proteins.

## Discussion

Natural products are a major source of pharmaceuticals and other bioactive chemicals. Continuous efforts have been paid to mining novel natural products to find solutions to antibiotic resistance as well as new bioactives that can be used as pharmaceuticals, agrochemicals, and nutraceuticals. However, heterologous expression of eukaryotic BGCs in genetically tractable hosts such as *S. cerevisiae* has met with several challenges, including the codon bias of tRNAs in heterologous hosts, inefficient cross-species recognition of regulatory elements, and intron splicing (Baral et al., 2018; Billingsley et al., 2016; Tsunematsu et al., 2013). Since eukaryotic BGCs usually contain over 10 genes ranging from tens to hundreds of kilobase, it is challenging to replace promoters and terminators and remove introns for each gene (Alberti et al., 2017; Harvey et al., 2018; Qiao et al., 2019). Therefore, characterizing cross-species recognition of transcriptional *cis*-elements, including the promoter, terminator, and intron, will significantly help efficient heterologous expression of target natural products.

In eukaryotic systems, each gene needs an individual promoter that can be recognized by RNA polymerases and transcription factors to initiate transcription (Curran et al., 2014), as well as an individual terminator to control the termination of transcription and mRNA half-life (Kuersten & Goodwin, 2003; Wang et al., 2019). The gene regulatory divergence between difference species has been reported (Lenz et al., 2020; Li & Fay, 2017), which demonstrated the complex expression profiles and the difficulties with cross-expression between different species. In this study, we evaluated cross-species expression efficiencies of promoters and terminators from *Penicillium* to yeast. Orotidine 5′-monophosphate decarboxylase, encoded by *URA3* in yeast and *pyrG* in *Penicillium*, is widely distributed in a plethora of organisms, and has been extensively used as a selection marker gene for uracil auxotroph (Lee et al., 2012; Yang et al., 2008). Therefore, we used orotidine 5′-monophosphate decarboxylase as a case study, and demonstrated that, although with reduced activities, all *pyrG* promoters from 14 *Penicillium* species can be recognized by yeast RNA polymerases and transcription factors, and could support yeast growth. Moreover, we are able to report that most *Penicillium* terminators further reduced the cross-species expression of *pyrG*, especially for *Penicillium* terminators from *P. digitatum, P. paneum, P. griseofulvum*, and *P. chrysogenum* (Fig. 1B), that recombinants with *Penicillium* promoters and ORFs (*pyrGp-pyrG-ScADH1t*) could grow on spotting plates; whereas recombinants with *Penicillium* promoters, terminators, and ORFs (*pyrGp-pyrG-pyrGt*) could not. This result demonstrates that besides promoters, terminators are also very important to allow efficient cross-species expression. In addition, we found that some recombinants grew well on the uracil dropout medium, but there were no detectable proteins during Western blot assay (Figs. 1, 3C, and 6B, C). It may be a minimal translation level of *pyrG* that could not even be detected in Western Blot is already enough to complement the cell growth (Jansen et al., 2012).

Intron splicing plays a crucial role in pre-mRNA processing. Eukaryotic genes are usually inserted with intron sequences, spliced through posttranscription (Herzel et al., 2017; Wilkinson et al., 2020). The number of introns per gene varies among different species, ranging from < 0.1 to 5.5 introns per gene in fungi (Sturm, 2006) and 0.04 intron per gene in *S. cerevisiae* (Kupfer et al., 2004; Qin et al., 2016). While *S. cerevisiae* has a molecular basis of intron splicing, the efficiency may not be enough to splice introns from distant species (Billingsley et al., 2016). Therefore, evaluation and engineering of yeast's capacity for cross-species splicing of fungi introns will continue to be the focus of heterologous expression. Unlike the intron-less *URA3* in yeast, *Penicillium pyrG* genes possess one short intron ranging in size from 36 to 72 bp. These intron sequences contain the canonical splice sites with GTAA at the 5′ end and CAG at the 3′ end. Even though recombinants either with *pyrG* genes or with *URA3* genes inserted with *pyrG* introns can grow on the uracil dropout medium, we were unable to detect target proteins using anti-Flag antibody in most recombinant strains by Western blot assay. These results suggested that a yeast native spliceosome cannot efficiently remove *Penicillium* introns. Similar results have been reported in yeast splicing introns from *Aspergillus. fumigatus* (DeNicola, 2018). We found that the *pyrG* introns have a relatively higher GC content ranging from 40–60% between the 5′-splice site and the branchpoint site compared with that of the second intron in *MATa1* (GC content around 22%). Higher GC content may also enhance the stability of RNA secondary structure (Zhang et al., 2011), and lead to inefficient splicing during the cross-species expression.

In this study, we cross-species expressed *pyrG* expression units from 14 *Penicillium* species in yeast. Our results suggested that the transcription *cis*-elements of *Penicillium pyrG*, including promoters, terminators, and intron sequences, can be recognized by the native machinery of *S. cerevisiae*, yet with poor activity. These findings shed light on the future engineering of *S. cerevisiae* for efficient and multiplexed expression of fungal expression units or even gene clusters for natural product discovery.

## Supplementary Material

kuab054_Supplemental_File
