# Supplementary material for: Characterization of cross-species transcription and splicing from Penicillium to Saccharomyces cerevisiae
Source: J Ind Microbiol Biotechnol. 2021 Aug 13;48(9-10):kuab054. doi: 10.1093/jimb/kuab054 (PMC8788760; doi:10.1093/jimb/kuab054)
Supplement: kuab054_Supplemental_File [file kuab054_supplemental_file.zip › Supplementary information Figure.docx]

Supplementary information


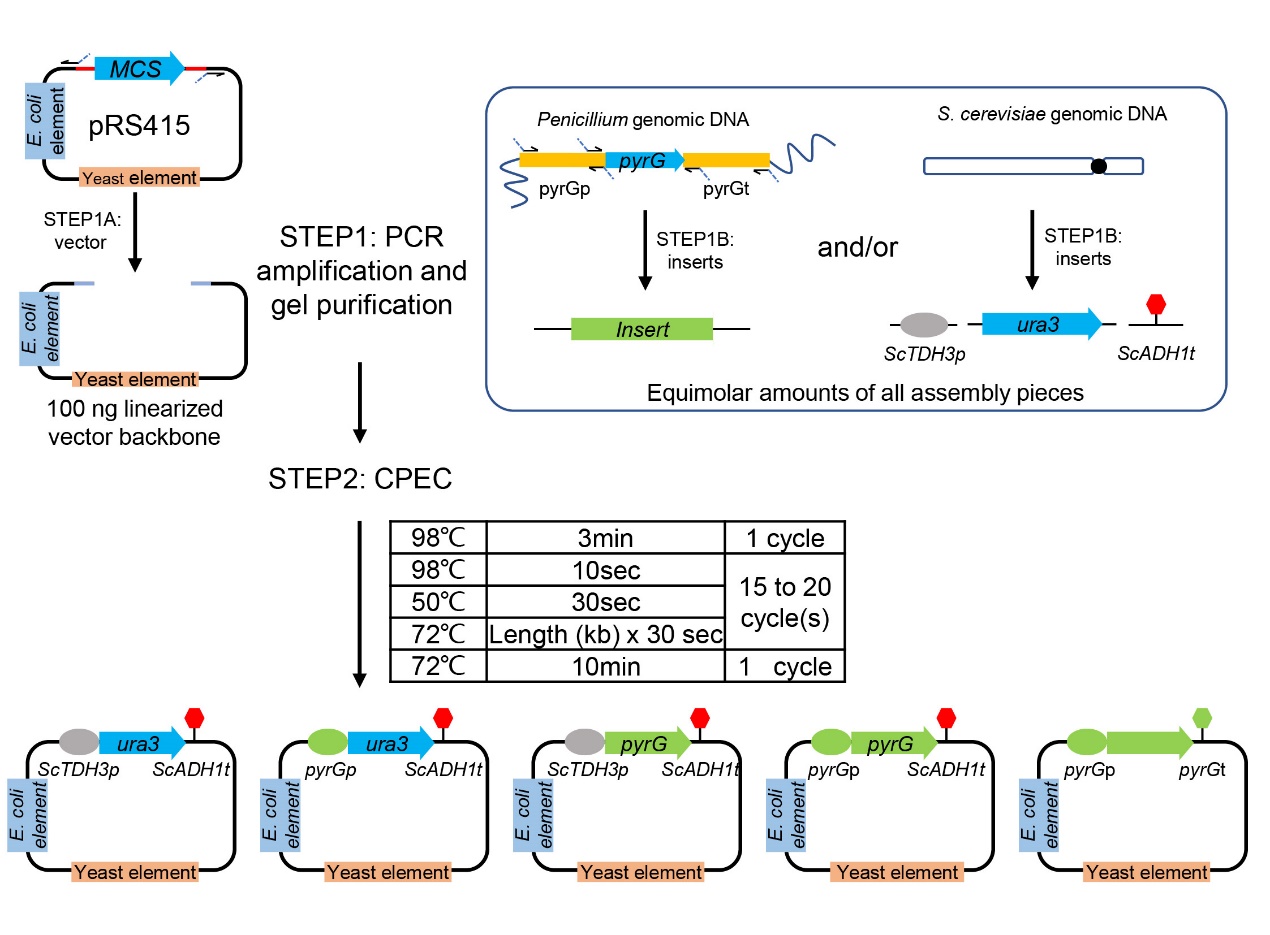


Fig S1.Schematic of plasmid construction


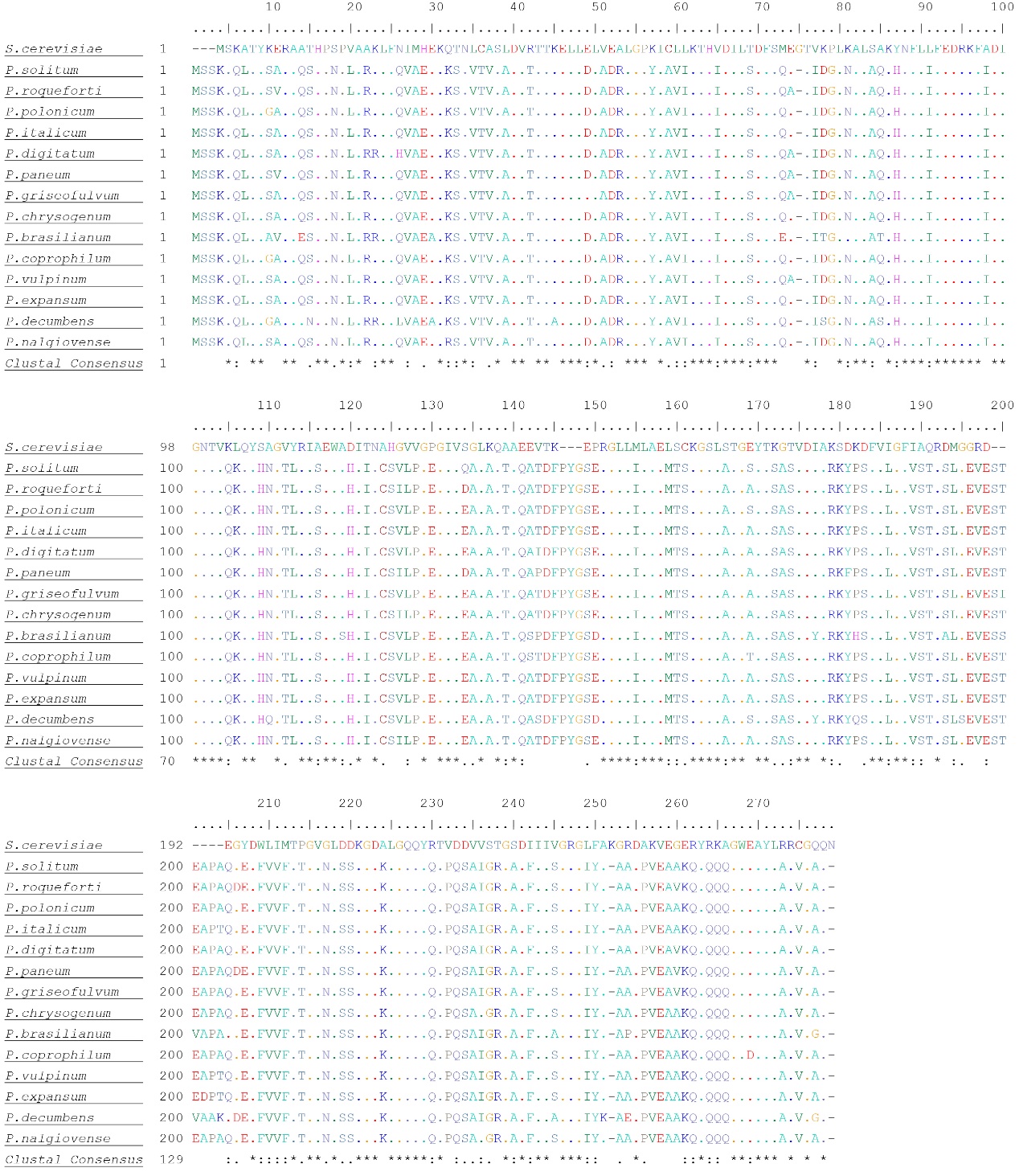


Fig. S2. Sequence alignment of orotidine 5'-monophosphate decarboxylases from fourteen Penicillium species. Alignment of amino acid sequences of orotidine 5'-monophosphate decarboxylases from S. cerevisiae and fourteen Penicillium species. Asterisk (*), fully conserved residues. Colon (:),conservation residues with strongly similar properties. Period (.), conservation residues with weakly similar properties.


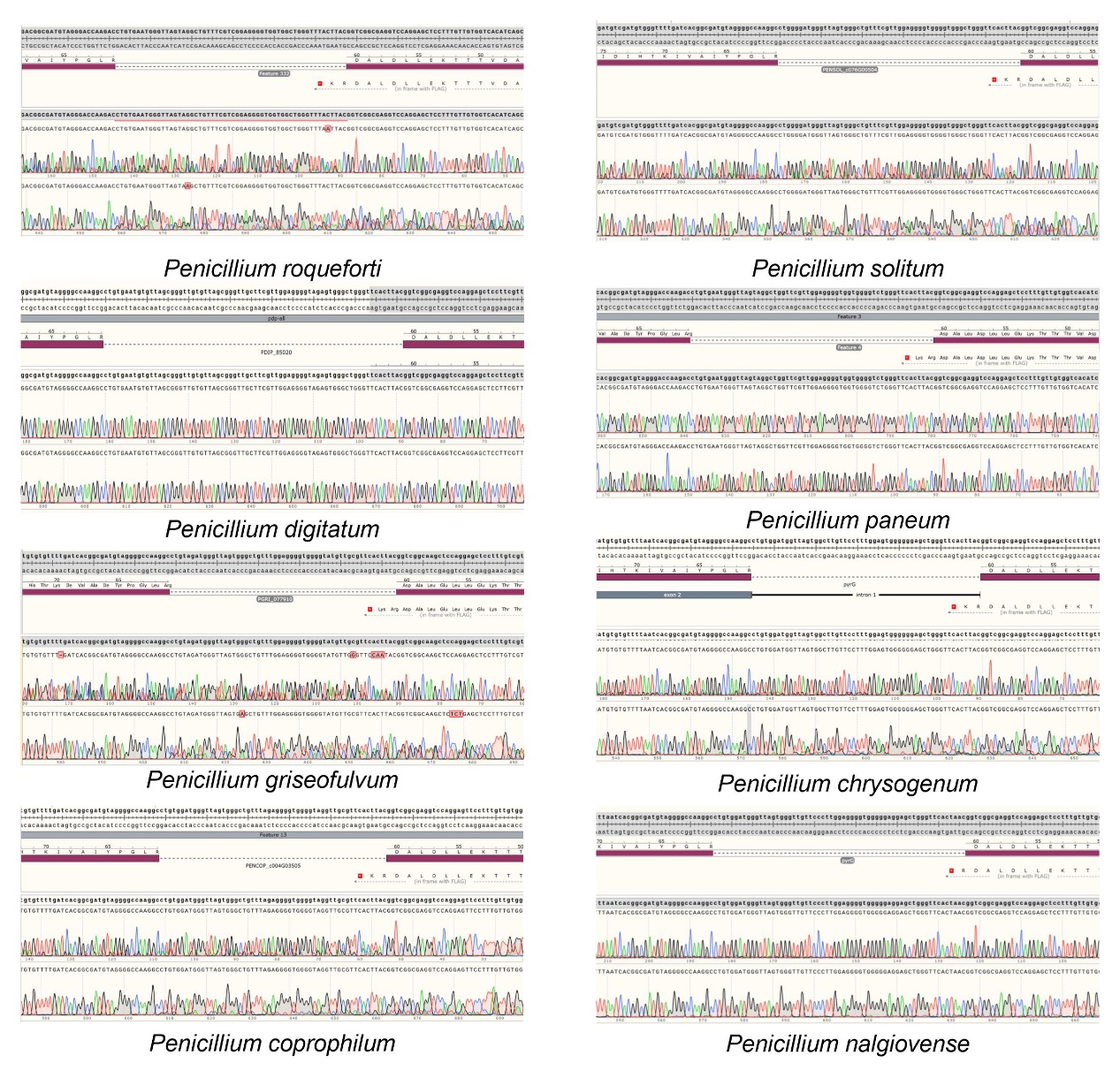


Fig S3. Sequence chromatograms of the cDNA sequence of yeast expressed pyrG genes from different origins
