# Supplementary material for: Characterization of cross-species transcription and splicing from Penicillium to Saccharomyces cerevisiae
Source: J Ind Microbiol Biotechnol. 2021 Aug 13;48(9-10):kuab054. doi: 10.1093/jimb/kuab054 (PMC8788760; doi:10.1093/jimb/kuab054)
Supplement: kuab054_Supplemental_File [file kuab054_supplemental_file.zip › Supplementary information Table S1.docx]

**Supplementary information**

**Supplementary table S1.** Plasmids, and primer sequences used in this study

| **Plasmids** | **Primer name** | **Primer sequence (5ʹ→3ʹ)** | **Templates** |
| --- | --- | --- | --- |
|  | **Primers for plasmid construction** | |  |
| pRS415-ScTDH3p-ScURA3-ScADH1t | ScTDH3p-F | attgtgagcggataacaatttcacaaaacgaatatatactagcgttgaatgttagcg | *S. cerevisiae* genomic DNA |
|  | ScTDH3p-R | acgttccttatatgtagctttcgacattttgtttgtttatgtgtgtttattcgaaac |  |
|  | ScURA3-F | aaacacacataaacaaacaaaatgtcgaaagctacatataaggaacgtgctgc |  |
|  | ScURA3-R | tcataagaaattcgcctcgaggcttacttataatacagttttttagttttgctggccgc |  |
|  | ScADH1t-F | caaaactaaaaaactgtattataagtaagcctcgaggcgaatttcttatgatttatga |  |
|  | ScADH1t-R | acagatgcgtaaggagaaaataccgcatcaggaacggtcgacccggtagaggtgtg |  |
|  | Backbone-F | cacacctctaccgggtcgaccgttcctgatgcggtattttctccttacgcatctgtg | pRS415 |
|  | Backbone-R | taacattcaacgctagtatatattcgttttgtgaaattgttatccgctcacaattccac |  |
| pRS415-pcspyrGp-ScURA3-ScADH1t | pcsP-P1 | TGGAATTGTGAGCGGATAACAATTTCACAgtgattgaagcttgaaattctctaatgtc | *P. rubens* genomic DNA |
|  | pcsP-P2 | acgttccttatatgtagctttcgacatggcggggttggcggggtgaagagatggttatg |  |
|  | pcsP-P3 | cataaccatctcttcaccccgccaaccccgccatgtcgaaagctacatataaggaacgt | pRS415-ScTDH3p-ScURA3-ScADH1t |
|  | pcsP-P4 | gacattagagaatttcaagcttcaatcacTGTGAAATTGTTATCCGCTCACAATTCCA |  |
| pRS415-ScTDH3p-pcspyrG-ScADH1t | pcsO-P1 | cttagtttcgaataaacacacataaacaaacaaacatctcttcaccccgccaaccccgc | *P. rubens* genomic DNA |
|  | PcsO-P2 | aaatcataaatcataagaaattcgcctcgaggcctatagtggaaggtggacgcacgctc |  |
|  | pcsO-P3 | gagcgtgcgtccaccttccactataggcctcgaggcgaatttcttatgatttatgattt | pRS415-ScTDH3p-ScURA3-ScADH1t |
|  | pcsO-P4 | gcggggttggcggggtgaagagatgtttgtttgtttatgtgtgtttattcgaaactaag |  |
| pRS415-pcspyrGp-pcspyrG-ScADH1t | pcsP-P1 | TGGAATTGTGAGCGGATAACAATTTCACAgtgattgaagcttgaaattctctaatgtc | *P. rubens* genomic DNA |
|  | pcsO-P2 | aaatcataaatcataagaaattcgcctcgaggcctatagtggaaggtggacgcacgctc |  |
|  | pcsO-P3 | gagcgtgcgtccaccttccactataggcctcgaggcgaatttcttatgatttatgattt | pRS415-ScTDH3p-ScURA3-ScADH1t |
|  | pcsP-P4 | gacattagagaatttcaagcttcaatcacTGTGAAATTGTTATCCGCTCACAATTCCA |  |
| pRS415-pcspyrGp-pcspyrG-pcspyrGt | pcsP-P1 | TGGAATTGTGAGCGGATAACAATTTCACAgtgattgaagcttgaaattctctaatgtc | *P. rubens* genomic DNA |
|  | pcsAll-P2 | CACAGATGCGTAAGGAGAAAATACCGCATCAGGaaagaacagtaggaactccgcacgc |  |
|  | pcsAll-P3 | gcgtgcggagttcctactgttctttCCTGATGCGGTATTTTCTCCTTACGCATCTGTG | pRS415-ScTDH3p-ScURA3-ScADH1t |
|  | pcsP-P4 | gacattagagaatttcaagcttcaatcacTGTGAAATTGTTATCCGCTCACAATTCCA |  |
| pRS415-pdpyrGp-ScURA3-ScADH1t | pdP-P1 | TGTGGAATTGTGAGCGGATAACAATTTCACAtcgaaacgtagggctcggaccggata | *P. decumbens* genomic DNA |
|  | pdP-P2 | cagcacgttccttatatgtagctttcgacatggtggatggcggggttggcgggat |  |
|  | pdP-P3 | atcccgccaaccccgccatccaccatgtcgaaagctacatataaggaacgtgctg | pRS415-ScTDH3p-ScURA3-ScADH1t |
|  | pdP-P4 | tatccggtccgagccctacgtttcgaTGTGAAATTGTTATCCGCTCACAATTCCACA |  |
| pRS415-ScTDH3p-pdpyrG-ScADH1t | pdO-P1 | cttcataatcccgccaaccccgccatccaccatgtcgtccaagtcccaattgaca | *P. decumbens* genomic DNA |
|  | pdO-P2 | tcataaatcataagaaattcgcctcgaggcaaagtgtccgtgtccgcaacggt |  |
|  | pdO-P3 | accgttgcggacacggacactttgcctcgaggcgaatttcttatgatttatga | pRS415-ScTDH3p-ScURA3-ScADH1t |
|  | pdO-P4 | tgtcaattgggacttggacgacatggtggatggcggggttggcgggattatgaag |  |
| pRS415-pdpyrGp-pdpyrG-ScADH1t | pdPO-P1 | GTGGAATTGTGAGCGGATAACAATTTCACAtcgaaacgtagggctcggaccggatac | *P. decumbens* genomic DNA |
|  | pdPO-P2 | tcataaatcataagaaattcgcctcgaggcaaagtgtccgtgtccgcaacggt |  |
|  | pdPO-P3 | accgttgcggacacggacactttgcctcgaggcgaatttcttatgatttatga | ScTDH3p-ura3-ADH1t |
|  | pdPO-P4 | gtatccggtccgagccctacgtttcgaTGTGAAATTGTTATCCGCTCACAATTCCAC |  |
| pRS415-pdpyrGp-pdpyrG-pdpyrGt | pdAll-P1 | GGAATTGTGAGCGGATAACAATTTCACAgccaaggctgagcagttcaactgcg | *P. decumbens* genomic DNA |
|  | pdAll-P2 | CGTAAGGAGAAAATACCGCATCAGGcgtcaagcatgggcttcttattggcttg |  |
|  | pdAll-P3 | caagccaataagaagcccatgcttgacgCCTGATGCGGTATTTTCTCCTTACG | pRS415-ScTDH3p-ScURA3-ScADH1t |
|  | pdAll-P4 | cgcagttgaactgctcagccttggcTGTGAAATTGTTATCCGCTCACAATTCC |  |
| pRS415-pdppyrGp-SCura3-ScADH1t | pdpP-P1 | TGGAATTGTGAGCGGATAACAATTTCACAgagcttgaagcctggggatcctctaaaatg | *P. digitatum* genomic DNA |
|  | pdpP-P2 | acgttccttatatgtagctttcgacatttcctacagtggaacgtggacagcacgctg |  |
|  | pdpP-P3 | cagcgtgctgtccacgttccactgtaggaaatgtcgaaagctacatataaggaacgt | pRS415-ScTDH3p-ScURA3-ScADH1t |
|  | pdpP-P4 | cattttagaggatccccaggcttcaagctcTGTGAAATTGTTATCCGCTCACAATTCCA |  |
| pRS415-ScTDH3p-pdppyrG-ScADH1t | pdpO-P1 | cttagtttcgaataaacacacataaacaaacaaaacacaagctaaatacacaacaatcg | *P. digitatum* genomic DNA |
|  | pdpO-P2 | cataaatcataagaaattcgcctcgaggctgcatcaccaagcacgttcctacagtg |  |
|  | pdpO-P3 | cactgtaggaacgtgcttggtgatgcagcctcgaggcgaatttcttatgatttatg | pRS415-ScTDH3p-ScURA3-ScADH1t |
|  | pdpO-P4 | cgattgttgtgtatttagcttgtgttttgtttgtttatgtgtgtttattcgaaactaag |  |
| pRS415-pdppyrGp-pdppyrG-ScADH1t | pdpP-P1 | TGGAATTGTGAGCGGATAACAATTTCACAgagcttgaagcctggggatcctctaaaatg | *P. digitatum* genomic DNA |
|  | pdpPO-P2 | cataaatcataagaaattcgcctcgaggcttcctacagtggaacgtggacagcacgct |  |
|  | pdpPO-P3 | agcgtgctgtccacgttccactgtaggaagcctcgaggcgaatttcttatgatttatg | pRS415-ScTDH3p-ScURA3-ScADH1t |
|  | pdpP-P4 | cattttagaggatccccaggcttcaagctcTGTGAAATTGTTATCCGCTCACAATTCCA |  |
| pRS415-pdppyrGp-pdppyrG-pdppyrGt | pdpAll-P1 | GTGGAATTGTGAGCGGATAACAATTTCACAgagcttgaagcctggggatcct | *P. digitatum* genomic DNA |
|  | pdpAll-P2 | CACAGATGCGTAAGGAGAAAATACCGCATCAGGcgtgaggtattcccaggagcat |  |
|  | pdpAll-P3 | atgctcctgggaatacctcacgCCTGATGCGGTATTTTCTCCTTACGCATCTGTG | pRS415-ScTDH3p-ScURA3-ScADH1t |
|  | pdpAll-P4 | aggatccccaggcttcaagctcTGTGAAATTGTTATCCGCTCACAATTCCAC |  |
| pRS415-pnpyrGp-ScURA3-ScADH1t | pnP-P1 | TGGAATTGTGAGCGGATAACAATTTCACAgaattcctcgcagacaatgctcttcaccct | *P. nalgiovense* genomic DNA |
|  | pnP-P2 | cgttccttatatgtagctttcgacatggcggggttggcggggtgaagagagggttat |  |
|  | pnP-P3 | ataaccctctcttcaccccgccaaccccgccatgtcgaaagctacatataaggaacg | pRS415-ScTDH3p-ScURA3-ScADH1t |
|  | pnP-P4 | agggtgaagagcattgtctgcgaggaattcTGTGAAATTGTTATCCGCTCACAATTCCA |  |
| pRS415-ScTDH3p-pnpyrG-ScADH1t | pnO-P1 | cttagtttcgaataaacacacataaacaaacaaacaaatacataaccctctcttcac | *P. nalgiovense* genomic DNA |
|  | pnO-P2 | atcataaatcataagaaattcgcctcgaggcatcaccacacatgtacctataatggaac |  |
|  | pnO-P3 | gttccattataggtacatgtgtggtgatgcctcgaggcgaatttcttatgatttatgat | pRS415-ScTDH3p-ScURA3-ScADH1t |
|  | pnO-P4 | gtgaagagagggttatgtatttgtttgtttgtttatgtgtgtttattcgaaactaag |  |
| pRS415-pnpyrGp-pnpyrG-ScADH1t | pnP-P1 | TGGAATTGTGAGCGGATAACAATTTCACAgaattcctcgcagacaatgctcttcaccct | *P. nalgiovense* genomic DNA |
|  | pnPO-P2 | cataaatcataagaaattcgcctcgaggcctattgcgcacccacgcgggccagatacg |  |
|  | pnPO-P3 | cgtatctggcccgcgtgggtgcgcaataggcctcgaggcgaatttcttatgatttatg | pRS415-ScTDH3p-ScURA3-ScADH1t |
|  | pnP-P4 | agggtgaagagcattgtctgcgaggaattcTGTGAAATTGTTATCCGCTCACAATTCCA |  |
| pRS415-pnpyrGp-pnpyrG-pnpyrGt | pnP-P1 | TGGAATTGTGAGCGGATAACAATTTCACAgaattcctcgcagacaatgctcttcaccct | *P. nalgiovense* genomic DNA |
|  | pnAll-P2 | CACAGATGCGTAAGGAGAAAATACCGCATCAGGgtcactcctatgaccagcgtagca |  |
|  | pnAll-P3 | tgctacgctggtcataggagtgacCCTGATGCGGTATTTTCTCCTTACGCATCTGTG | pRS415-ScTDH3p-ScURA3-ScADH1t |
|  | pnP-P4 | agggtgaagagcattgtctgcgaggaattcTGTGAAATTGTTATCCGCTCACAATTCCA |  |
| pRS415-PropyrGp-*ScURA3*-ScADH1t | proP-P1 | GAATTGTGAGCGGATAACAATTTCACAtacggagtagatgaCTGGGCGGAACGGAC | *P. roqueforti* genomic DNA |
|  | proP-P2 | CATATCAACCCACCCCGCCAACCCCGCCatgtcgaaagctacatataaggaacgtgctg |  |
|  | proP-P3 | cagcacgttccttatatgtagctttcgacatGGCGGGGTTGGCGGGGTGGGTTGATATG | pRS415-ScTDH3p-ScURA3-ScADH1t |
|  | proP-P4 | GTCCGTTCCGCCCAGtcatctactccgtaTGTGAAATTGTTATCCGCTCACAATTC |  |
| pRS415-ScTDH3p-PropyrG-ScADH1t | proO-P1 | aataaacacacataaacaaacaaAATGTCGTCCAAGTCGCAATTGACCTATAG | *P. roqueforti* genomic DNA |
|  | proO-P2 | cataaatcataagaaattcgcctcgaggcCTATTGCGCACCCACACGGGCCAGATAC |  |
|  | proO-P3 | GTATCTGGCCCGTGTGGGTGCGCAATAGgcctcgaggcgaatttcttatgatttatg | pRS415-ScTDH3p-ScURA3-ScADH1t |
|  | proO-P4 | CTATAGGTCAATTGCGACTTGGACGACATtttgtttgtttatgtgtgtttatt |  |
| pRS415-PropyrGp-PropyrG-ScADH1t | proP-P1 | GAATTGTGAGCGGATAACAATTTCACAtacggagtagatgaCTGGGCGGAACGGAC | *P. roqueforti* genomic DNA |
|  | proO-P2 | cataaatcataagaaattcgcctcgaggcCTATTGCGCACCCACACGGGCCAGATAC |  |
|  | proO-P3 | GTATCTGGCCCGTGTGGGTGCGCAATAGgcctcgaggcgaatttcttatgatttatg | pRS415-ScTDH3p-ScURA3-ScADH1t |
|  | proP-P4 | GTCCGTTCCGCCCAGtcatctactccgtaTGTGAAATTGTTATCCGCTCACAATTC |  |
| pRS415-PropyrGp-PropyrG-PropyrGt | proP-P1 | CATATCAACCCACCCCGCCAACCCCGCCatgtcgaaagctacatataaggaacgtgctg | *P. roqueforti* genomic DNA |
|  | proAll-P2 | GATGCGTAAGGAGAAAATACCGCATCAGGACTATAAGCAATACCAAGGCAATG |  |
|  | proAll-P3 | CATTGCCTTGGTATTGCTTATAGTCCTGATGCGGTATTTTCTCCTTACGCATC | pRS415-ScTDH3p-ScURA3-ScADH1t |
|  | proP-P4 | GTCCGTTCCGCCCAGtcatctactccgtaTGTGAAATTGTTATCCGCTCACAATTC |  |
| pRS415-psopyrGp-ScURA3-ScADH1t | psoP-P1 | GGAATTGTGAGCGGATAACAATTTCACActcgaacaagccccagggtaagggccgcatg | *P. solitum* genomic DNA |
|  | psoP-P2 | cacgttccttatatgtagctttcgacatggtggggttggcggggtgaagaggtggttg |  |
|  | psoP-P3 | caaccacctcttcaccccgccaaccccaccatgtcgaaagctacatataaggaacgtg | pRS415-ScTDH3p-ScURA3-ScADH1t |
|  | psoP-P4 | catgcggcccttaccctggggcttgttcgagTGTGAAATTGTTATCCGCTCACAATTCC |  |
| pRS415-ScTDH3p-psopyrG-ScADH1t | psoO-P1 | gaataaacacacataaacaaacaaaatgtcgtccaagtcgcaattgacctatagcgctc | *P. solitum* genomic DNA |
|  | psoO-P2 | cataaatcataagaaattcgcctcgaggcctattgtgcgcccacgcgggccagatacgc |  |
|  | psoO-P3 | gcgtatctggcccgcgtgggcgcacaataggcctcgaggcgaatttcttatgatttatg | pRS415-ScTDH3p-ScURA3-ScADH1t |
|  | psoO-P4 | gagcgctataggtcaattgcgacttggacgacattttgtttgtttatgtgtgtttattc |  |
| pRS415-psopyrGp-psopyrG-ScADH1t | psoP-P1 | GGAATTGTGAGCGGATAACAATTTCACActcgaacaagccccagggtaagggccgcatg | *P. solitum* genomic DNA |
|  | psoO-P2 | cataaatcataagaaattcgcctcgaggcctattgtgcgcccacgcgggccagatacgc |  |
|  | psoO-P3 | gcgtatctggcccgcgtgggcgcacaataggcctcgaggcgaatttcttatgatttatg | pRS415-ScTDH3p-ScURA3-ScADH1t |
|  | psoP-P4 | catgcggcccttaccctggggcttgttcgagTGTGAAATTGTTATCCGCTCACAATTCC |  |
| pRS415-psopyrGp-psopyrG-psopyrGt | psoP-P1 | GGAATTGTGAGCGGATAACAATTTCACActcgaacaagccccagggtaagggccgcatg | *P. solitum* genomic DNA |
|  | PsoAll-P2 | GTAAGGAGAAAATACCGCATCAGGgccactaaagtacaaagtactttctaccatc |  |
|  | psoAll-P3 | gatggtagaaagtactttgtactttagtggcCCTGATGCGGTATTTTCTCCTTAC | pRS415-ScTDH3p-ScURA3-ScADH1t |
|  | psoP-P4 | catgcggcccttaccctggggcttgttcgagTGTGAAATTGTTATCCGCTCACAATTCC |  |
| pRS415-PbpyrGp-ScURA3-ScADH1t | pbP-P1 | GTGAGCGGATAACAATTTCACAccgcttgtgtgactagacgttgag | *P. brasilianum* genomic DNA |
|  | pbP-P2 | gttccttatatgtagctttcgacatgatggcggggttggcgggggagtag |  |
|  | pbP-P3 | gatatctactcccccgccaaccccgccatcatgtcgaaagctacatataa | pRS415-ScTDH3p-ScURA3-ScADH1t |
|  | pbP-P4 | caacgtctagtcacacaagcggTGTGAAATTGTTATCCGCTCACAATTCC |  |
| pRS415-ScTDH3p-PbpyrG-ScADH1t | pbO-P1 | gaataaacacacataaacaaacaaacaggccctattagcacttgcaatcgtatctcctg | *P. brasilianum* genomic DNA |
|  | pbO-P2 | cataaatcataagaaattcgcctcgaggcacgtacgtcatagtcaattctatatgt |  |
|  | pbO-P3 | gattaaaaacatatagaattgactatgacgtacgtgcctcgaggcgaatttcttatg | pRS415-ScTDH3p-ScURA3-ScADH1t |
|  | pbO-P4 | gatacgattgcaagtgctaatagggcctgtttgtttgtttatgtgtgtttattcgaaac |  |
| pRS415-PbpyrGp-PbpyrG-ScADH1t | pbP-P1 | GTGAGCGGATAACAATTTCACAccgcttgtgtgactagacgttgag | *P. brasilianum* genomic DNA |
|  | pbO-P2 | cataaatcataagaaattcgcctcgaggcacgtacgtcatagtcaattctatatgt |  |
|  | pbO-P3 | gattaaaaacatatagaattgactatgacgtacgtgcctcgaggcgaatttcttatg | pRS415-ScTDH3p-ScURA3-ScADH1t |
|  | pbP-P4 | caacgtctagtcacacaagcggTGTGAAATTGTTATCCGCTCACAATTCC |  |
| pRS415-PbpyrGp-PbpyrG-PbpyrGt | pbP-P1 | GTGAGCGGATAACAATTTCACAccgcttgtgtgactagacgttgag | *P. brasilianum* genomic DNA |
|  | pbAll-P2 | gtagtgcttggggagattaggtcttggcgcttgattttgggagccttgcaaagctg |  |
|  | pbAll-P3 | ttgcaaggctcccaaaatcaagcgccaagacctaatctccccaagcactacgccaag | pRS415-ScTDH3p-ScURA3-ScADH1t |
|  | pbP-P4 | caacgtctagtcacacaagcggTGTGAAATTGTTATCCGCTCACAATTCC |  |
| pRS415-PpapyrGp-ScURA3-ScADH1t | ppaP-P1 | GTGAGCGGATAACAATTTCACAtacttcttatggcacctccttaactagttataactta | *P. paneum* genomic DNA |
|  | ppaP-P2 | agcacgttccttatatgtagctttcgacatggcgtggttggtggagtgggttgatatgt |  |
|  | ppaP-P3 | tcaacccactccaccaaccacgccatgtcgaaagctacatataaggaacgtgctgctac | pRS415-ScTDH3p-ScURA3-ScADH1t |
|  | ppaP-P4 | taactagttaaggaggtgccataagaagtaTGTGAAATTGTTATCCGCTCACAATTCCA |  |
| pRS415-ScTDH3p-PpapyrG-ScADH1t | ppaO-P1 | gaataaacacacataaacaaacaaaatttgtcgggcattttcaatttcattcgcttctc | *P. paneum* genomic DNA |
|  | ppaO-P2 | cataaatcataagaaattcgcctcgaggcagcatcaagatgcaaaaaggttatatagca |  |
|  | ppaO-P3 | tataacctttttgcatcttgatgctgcctcgaggcgaatttcttatgatttatgatttt | pRS415-ScTDH3p-ScURA3-ScADH1t |
|  | ppaO-P4 | gcgaatgaaattgaaaatgcccgacaaattttgtttgtttatgtgtgtttattcgaaac |  |
| pRS415-PpapyrGp-PpapyrG-ScADH1t | ppaPO-P1 | TTGTGAGCGGATAACAATTTCACAtacttcttatggcacctccttaactagttataact | *P. paneum* genomic DNA |
|  | ppaO-P2 | cataaatcataagaaattcgcctcgaggcagcatcaagatgcaaaaaggttatatagca |  |
|  | ppaO-P3 | tataacctttttgcatcttgatgctgcctcgaggcgaatttcttatgatttatgatttt | pRS415-ScTDH3p-ScURA3-ScADH1t |
|  | ppaPO-P4 | taactagttaaggaggtgccataagaagtaTGTGAAATTGTTATCCGCTCACAATTCCA |  |
| pRS415-PpapyrGp-PpapyrG-PpapyrGt | ppaPO-P1 | TTGTGAGCGGATAACAATTTCACAtacttcttatggcacctccttaactagttataact | *P. paneum* genomic DNA |
|  | ppaAll-P2 | GATGCGTAAGGAGAAAATACCGCATCAGGtaatgtaaaggttaattgcttggtgtgtac |  |
|  | ppaAll-P3 | cacatgtacacaccaagcaattaacctttacattaCCTGATGCGGTATTTTCTCCTTAC | pRS415-ScTDH3p-ScURA3-ScADH1t |
|  | ppaPO-P4 | taactagttaaggaggtgccataagaagtaTGTGAAATTGTTATCCGCTCACAATTCCA |  |
| pRS415-PipyrGp-PipyrG –PipyrGt | ITAP-P1 | ATTGTGAGCGGATAACAATTTCACAtgggttttattccgcgttatgttcctttac | *P. italicum* genomic DNA |
|  | ITAAll-P2 | GATGCGTAAGGAGAAAATACCGCATCAGGtattacaatacataacaaatcgacaagcg |  |
|  | ITAAll-P3 | gtatacgcttgtcgatttgttatgtattgtaataCCTGATGCGGTATTTTCTCCTTAC | pRS415-ScTDH3p-ScURA3-ScADH1t |
|  | ITAP-P4 | ggaacataacgcggaataaaacccaTGTGAAATTGTTATCCGCTCACAATTCCAC |  |
| pRS415-PipyrGp-PipyrG –ScADH1t | ITAP-P1 | ATTGTGAGCGGATAACAATTTCACAtgggttttattccgcgttatgttcctttac | *P. italicum* genomic DNA |
|  | ITAO-P2 | cataagaaattcgcctcgaggcgccaaaaggttatatagtatgccatcaag |  |
|  | ITAO-P3 | tggcatactatataaccttttggcgcctcgaggcgaatttcttatgatttatg | pRS415-ScTDH3p-ScURA3-ScADH1t |
|  | ITAP-P4 | ggaacataacgcggaataaaacccaTGTGAAATTGTTATCCGCTCACAATTCCAC |  |
| pRS415-PipyrGp-ScURA3-ScADH1t | ITAP-P1 | ATTGTGAGCGGATAACAATTTCACAtgggttttattccgcgttatgttcctttac | *P. italicum* genomic DNA |
|  | ITAP-P2 | cacgttccttatatgtagctttcgacatggcagggttggcggggttggcggggtgaaga |  |
|  | ITAP-P3 | ccgccaaccccgccaaccctgccatgtcgaaagctacatataaggaacgtgctgctac | pRS415-ScTDH3p-ScURA3-ScADH1t |
|  | ITAP-P4 | ggaacataacgcggaataaaacccaTGTGAAATTGTTATCCGCTCACAATTCCAC |  |
| pRS415-ScTDH3p-PipyrG-ScADH1t | ITAO-P1 | gtttcgaataaacacacataaacaaacaaacatttgattcacttccctcttgaaatc | *P. italicum* genomic DNA |
|  | ITAO-P2 | cataagaaattcgcctcgaggcgccaaaaggttatatagtatgccatcaag |  |
|  | ITAO-P3 | tggcatactatataaccttttggcgcctcgaggcgaatttcttatgatttatg | pRS415-ScTDH3p-ScURA3-ScADH1t |
|  | ITAO-P4 | gtaggatttcaagagggaagtgaatcaaatgtttgtttgtttatgtgtgtttattcg |  |
| pRS415-PpopyrGp-ScURA3-ScADH1t | polP-P1 | TTGTGAGCGGATAACAATTTCACAgaagtacccggagttggctgctggtctgggcgctc | *P. polonicum* genomic DNA |
|  | polP-P2 | tccttatatgtagctttcgacatggtggggttggcggggtgaagaggtggttgtttatg |  |
|  | polP-P3 | caaccacctcttcaccccgccaaccccaccatgtcgaaagctacatataaggaacgtgc | pRS415-ScTDH3p-ScURA3-ScADH1t |
|  | polP-P4 | ccagaccagcagccaactccgggtacttcTGTGAAATTGTTATCCGCTCACAATTCCAC |  |
| pRS415-ScTDH3p-PpopyrG-ScADH1t | polO-P1 | ataaacacacataaacaaacaaaaggcatttgtcgtttgctccatttccctaaaatctc | *P. polonicum* genomic DNA |
|  | polO-P2 | tcataagaaattcgcctcgaggcaggctgcaaaaatatatcacgccatcaaatgtatat |  |
|  | polO-P3 | atttgatggcgtgatatatttttgcagcctgcctcgaggcgaatttcttatgatttatg | pRS415-ScTDH3p-ScURA3-ScADH1t |
|  | polO-P4 | ttagggaaatggagcaaacgacaaatgccttttgtttgtttatgtgtgtttattcgaaa |  |
| pRS415-PpopyrGp-PpopyrG-ScADH1t | polP-P1 | TTGTGAGCGGATAACAATTTCACAgaagtacccggagttggctgctggtctgggcgctc | *P. polonicum* genomic DNA |
|  | polO-P2 | tcataagaaattcgcctcgaggcaggctgcaaaaatatatcacgccatcaaatgtatat |  |
|  | polO-P3 | atttgatggcgtgatatatttttgcagcctgcctcgaggcgaatttcttatgatttatg | pRS415-ScTDH3p-ScURA3-ScADH1t |
|  | polP-P4 | ccagaccagcagccaactccgggtacttcTGTGAAATTGTTATCCGCTCACAATTCCAC |  |
| pRS415-PpopyrGp-PpopyrG-PpopyrGt | polP-P1 | TTGTGAGCGGATAACAATTTCACAgaagtacccggagttggctgctggtctgggcgctc | *P. polonicum* genomic DNA |
|  | polAll-P2 | GATGCGTAAGGAGAAAATACCGCATCAGGaagcgtggtaggtaggtgctggcatataac |  |
|  | polAll-P3 | tgccagcacctacctaccacgcttCCTGATGCGGTATTTTCTCCTTACGCATCTGTGCG | pRS415-ScTDH3p-ScURA3-ScADH1t |
|  | polP-P4 | ccagaccagcagccaactccgggtacttcTGTGAAATTGTTATCCGCTCACAATTCCAC |  |
| pRS415-PvpyrGp-ScURA3-ScADH1t | vulP-P1 | TTGTGAGCGGATAACAATTTCACAcaagccgcagggtcagggccgtctggtgggctcg | *P. vulpinum* genomic DNA |
|  | vulP-P2 | cttatatgtagctttcgacatggcgggattgggctgaagaggcggttgttatgtatttg |  |
|  | vulP-P3 | taacaaccgcctcttcagcccaatcccgccatgtcgaaagctacatataaggaacgtgc | pRS415-ScTDH3p-ScURA3-ScADH1t |
|  | vulP-P4 | ccaccagacggccctgaccctgcggcttgTGTGAAATTGTTATCCGCTCACAATTCCAC |  |
| pRS415-ScTDH3p-PvpyrG-ScADH1t | vulO-P1 | cgaataaacacacataaacaaacaaatccgtcatttgattaacctcaatcctcttg | *P. vulpinum* genomic DNA |
|  | vulO-P2 | tcataagaaattcgcctcgaggcgcaaaaaggttatgttgcacgccatcaaatatag |  |
|  | vulO-P3 | atttgatggcgtgcaacataacctttttgcgcctcgaggcgaatttcttatgatttatg | pRS415-ScTDH3p-ScURA3-ScADH1t |
|  | vulO-P4 | aggattgaggttaatcaaatgacggatttgtttgtttatgtgtgtttattcgaaac |  |
| pRS415-PvpyrGp-PvpyrG-ScADH1t | vulP-P1 | TTGTGAGCGGATAACAATTTCACAcaagccgcagggtcagggccgtctggtgggctcg | *P. vulpinum* genomic DNA |
|  | vulO-P2 | tcataagaaattcgcctcgaggcgcaaaaaggttatgttgcacgccatcaaatatag |  |
|  | vulO-P3 | atttgatggcgtgcaacataacctttttgcgcctcgaggcgaatttcttatgatttatg | pRS415-ScTDH3p-ScURA3-ScADH1t |
|  | vulP-P4 | ccaccagacggccctgaccctgcggcttgTGTGAAATTGTTATCCGCTCACAATTCCAC |  |
| pRS415-PvpyrGp-PvpyrG-PvpyrGt | vulP-P1 | TTGTGAGCGGATAACAATTTCACAcaagccgcagggtcagggccgtctggtgggctcg | *P. vulpinum* genomic DNA |
|  | vulAll-P2 | TAAGGAGAAAATACCGCATCAGGtgtcggtgtgggattcgacagatcatagcacgcgtg |  |
|  | vulAll-P3 | tgctatgatctgtcgaatcccacaccgacaCCTGATGCGGTATTTTCTCCTTACGCATC | pRS415-ScTDH3p-ScURA3-ScADH1t |
|  | vulP-P4 | ccaccagacggccctgaccctgcggcttgTGTGAAATTGTTATCCGCTCACAATTCCAC |  |
| pRS415-PcpyrGp-ScURA3-ScADH1t | pcP-P1 | TGTGAGCGGATAACAATTTCACAtcttcaccctggtcgccggtctgaagtacccagag | *P. coprophilum* genomic DNA |
|  | pcP-P2 | ccttatatgtagctttcgacatgacgcggtcaggtgaagagacgggtctgtatttgtac |  |
|  | pcP-P3 | tacagacccgtctcttcacctgaccgcgtcatgtcgaaagctacatataaggaacgtgc | pRS415-ScTDH3p-ScURA3-ScADH1t |
|  | pcP-P4 | ggtacttcagaccggcgaccagggtgaagaTGTGAAATTGTTATCCGCTCACAATTCC |  |
| pRS415-ScTDH3p-PcpyrG-ScADH1t | pcO-P1 | aataaacacacataaacaaacaaatttgaatctgttcattcactttcctcctttcctcc | *P. coprophilum* genomic DNA |
|  | pcO-P2 | tcataagaaattcgcctcgaggccaaaatggagatatggcaagccatcaaatctagaac |  |
|  | pcO-P3 | gatttgatggcttgccatatctccattttggcctcgaggcgaatttcttatgatttatg | pRS415-ScTDH3p-ScURA3-ScADH1t |
|  | pcO-P4 | aaggaggaaagtgaatgaacagattcaaatttgtttgtttatgtgtgtttattcgaaac |  |
| pRS415-PcpyrGp-PcpyrG-ScADH1t | pcP-P1 | TGTGAGCGGATAACAATTTCACAtcttcaccctggtcgccggtctgaagtacccagag | *P. coprophilum* genomic DNA |
|  | pcO-P2 | tcataagaaattcgcctcgaggccaaaatggagatatggcaagccatcaaatctagaac |  |
|  | pcO-P3 | gatttgatggcttgccatatctccattttggcctcgaggcgaatttcttatgatttatg | pRS415-ScTDH3p-ScURA3-ScADH1t |
|  | pcP-P4 | ggtacttcagaccggcgaccagggtgaagaTGTGAAATTGTTATCCGCTCACAATTCC |  |
| pRS415-PcpyrGp-PcpyrG-PcpyrGt | pcP-P1 | TGTGAGCGGATAACAATTTCACAtcttcaccctggtcgccggtctgaagtacccagag | *P. coprophilum* genomic DNA |
|  | pcAll-P2 | GTAAGGAGAAAATACCGCATCAGGgttgttcaatatgaagttaccagtataatgcagac |  |
|  | pcAll-P3 | cattatactggtaacttcatattgaacaacCCTGATGCGGTATTTTCTCCTTACGCATC | pRS415-ScTDH3p-ScURA3-ScADH1t |
|  | pcP-P4 | ggtacttcagaccggcgaccagggtgaagaTGTGAAATTGTTATCCGCTCACAATTCC |  |
| pRS415-PgpyrGp-ScURA3-ScADH1t | pgP-P1 | GTGTGGAATTGTGAGCGGATAACAATTTCACAatcttgtaataacctgaagtg | *P. griseofulvum* genomic DNA |
|  | pgP-P2 | cttatatgtagctttcgacatggcggggccgaagagatgtgtatttggt |  |
|  | pgP-P3 | accaaatacacatctcttcggccccgccatgtcgaaagctacatataag | pRS415-ScTDH3p-ScURA3-ScADH1t |
|  | pgP-P4 | cacttcaggttattacaagatTGTGAAATTGTTATCCGCTCACAATTCCACAC |  |
| pRS415-ScTDH3p-PgpyrG-ScADH1t | pgO-P1 | cgaataaacacacataaacaaacaaacctgaggtcattcaatttcctc | *P. griseofulvum* genomic DNA |
|  | pgO-P2 | gaaattcgcctcgaggccattatgcatcacgcatgcaactataatag |  |
|  | pgO-P3 | ctattatagttgcatgcgtgatgcataatggcctcgaggcgaatttc | pRS415-ScTDH3p-ScURA3-ScADH1t |
|  | pgO-P4 | gaggaaattgaatgacctcaggtttgtttgtttatgtgtgtttattcg |  |
| pRS415-PgpyrGp-PgpyrG-ScADH1t | pgP-P1 | GTGTGGAATTGTGAGCGGATAACAATTTCACAatcttgtaataacctgaagtg | *P. griseofulvum* genomic DNA |
|  | pgO-P2 | gaaattcgcctcgaggccattatgcatcacgcatgcaactataatag |  |
|  | pgO-P3 | ctattatagttgcatgcgtgatgcataatggcctcgaggcgaatttc | pRS415-ScTDH3p-ScURA3-ScADH1t |
|  | pgP-P4 | cacttcaggttattacaagatTGTGAAATTGTTATCCGCTCACAATTCCACAC |  |
| pRS415-PgpyrGp-PgpyrG-PgpyrGt | pgP-P1 | GTGTGGAATTGTGAGCGGATAACAATTTCACAatcttgtaataacctgaagtg | *P. griseofulvum* genomic DNA |
|  | pgAll-P2 | GATGCGTAAGGAGAAAATACCGCATCAGGttcaatatagggctagatacttggtg |  |
|  | pgAll-P3 | caccaagtatctagccctatattgaaCCTGATGCGGTATTTTCTCCTTACGCATC | pRS415-ScTDH3p-ScURA3-ScADH1t |
|  | pgP-P4 | cacttcaggttattacaagatTGTGAAATTGTTATCCGCTCACAATTCCACAC |  |
| pRS415-PepyrGp-ScURA3-ScADH1t | peP-P1 | GTTGTGTGGAATTGTGAGCGGATAACAATTTCACAatgtgagcttggaatcctctaa | *P. expansum* genomic DNA |
|  | peP-P2 | cgttccttatatgtagctttcgacatggtggggttggcggggtgaagagcggttg |  |
|  | peP-P3 | gctcttcaccccgccaaccccaccatgtcgaaagctacatataaggaacgtgctg | pRS415-ScTDH3p-ScURA3-ScADH1t |
|  | peP-P4 | gacattttagaggattccaagctcacatTGTGAAATTGTTATCCGCTCACAATTCCAC |  |
| pRS415-ScTDH3p-PepyrG-ScADH1t | peO-P1 | tagtttcgaataaacacacataaacaaacaaacatctgtcgtttaattcacttccatc | *P. expansum* genomic DNA |
|  | peO-P2 | cataaatcataagaaattcgcctcgaggcagaagattcatatagcatgccatc |  |
|  | peO-P3 | tatttgatggcatgctatatgaatcttctgcctcgaggcgaatttcttatg | pRS415-ScTDH3p-ScURA3-ScADH1t |
|  | peO-P4 | cagaggatggaagtgaattaaacgacagatgtttgtttgtttatgtgtgtttattcg |  |
| pRS415-PepyrGp-PepyrG-ScADH1t | peP-P1 | GTTGTGTGGAATTGTGAGCGGATAACAATTTCACAatgtgagcttggaatcctctaa | *P. expansum* genomic DNA |
|  | pePO-P2 | cataaatcataagaaattcgcctcgaggcagaagattcatatagcatgccatc |  |
|  | pePO-P3 | tatttgatggcatgctatatgaatcttctgcctcgaggcgaatttcttatg | pRS415-ScTDH3p-ScURA3-ScADH1t |
|  | peP-P4 | gacattttagaggattccaagctcacatTGTGAAATTGTTATCCGCTCACAATTCCAC |  |
| pRS415-PepyrGp-PepyrG-PepyrGt | peP-P1 | GTTGTGTGGAATTGTGAGCGGATAACAATTTCACAatgtgagcttggaatcctctaa | *P. expansum* genomic DNA |
|  | peAll-P2 | CACAGATGCGTAAGGAGAAAATACCGCATCAGGagaagattcatatagcatgccatc |  |
|  | peAll-P3 | tatttgatggcatgctatatgaatcttctCCTGATGCGGTATTTTCTCCTTACGCATC | pRS415-ScTDH3p-ScURA3-ScADH1t |
|  | peP-P4 | gacattttagaggattccaagctcacatTGTGAAATTGTTATCCGCTCACAATTCCAC |  |
|  |  |  |  |
|  | **Primers for FLAG-TAG addition** | |  |
| proP-FLAG TAG | URA3TAG1 | atgGATTACAAGGATGACGACGATAAGtcgaaagctacatataaggaacg | pRS415-PropyrGp-*ScURA3*-ScADH1t |
|  | pyrGTAG2 | CAAGATAGTGGCGATAGGGTCAAC |  |
|  | pyrGTAG3 | GATATCATCTCCGATGAAGCCTCCG |  |
|  | proTAG4 | CTTATCGTCGTCATCCTTGTAATCCATGGCGGGGTTGGCGGGGTGGGTTGA |  |
| proO-FLAG TAG | pyrGtag1 | ATGGATTACAAGGATGACGACGATAAGTCGTCCAAGTCGCAATTGAC | pRS415-ScTDH3p-PropyrG-ScADH1t |
|  | pyrGTAG2 | CAAGATAGTGGCGATAGGGTCAAC |  |
|  | pyrGTAG3 | GATATCATCTCCGATGAAGCCTCCG |  |
|  | tdhTAG4 | CTTATCGTCGTCATCCTTGTAATCcattttgtttgtttatgtgtgtttattcg |  |
| proPO-FLAG TAG | pyrGtag1 | ATGGATTACAAGGATGACGACGATAAGTCGTCCAAGTCGCAATTGAC | pRS415-PropyrGp-PropyrG-ScADH1t |
|  | pyrGTAG2 | CAAGATAGTGGCGATAGGGTCAAC |  |
|  | pyrGTAG3 | GATATCATCTCCGATGAAGCCTCCG |  |
|  | proTAG4 | CTTATCGTCGTCATCCTTGTAATCCATGGCGGGGTTGGCGGGGTGGGTTGA |  |
| proAll-FLAG TAG | pyrGtag1 | ATGGATTACAAGGATGACGACGATAAGTCGTCCAAGTCGCAATTGAC | pRS415-PropyrGp-PropyrG-PropyrGt |
|  | pyrGTAG2 | CAAGATAGTGGCGATAGGGTCAAC |  |
|  | pyrGTAG3 | GATATCATCTCCGATGAAGCCTCCG |  |
|  | proTAG4 | CTTATCGTCGTCATCCTTGTAATCCATGGCGGGGTTGGCGGGGTGGGTTGA |  |
| pnP-FLAG TAG | URA3TAG1 | atgGATTACAAGGATGACGACGATAAGtcgaaagctacatataaggaacg | pRS415-pnpyrGp-ScURA3-ScADH1t |
|  | pyrGTAG2 | CAAGATAGTGGCGATAGGGTCAAC |  |
|  | pyrGTAG3 | GATATCATCTCCGATGAAGCCTCCG |  |
|  | pnTAG4 | CTTATCGTCGTCATCCTTGTAATCcatggcggggttggcggggtgaagag |  |
| pnO-FLAG TAG | pyrGtag1 | ATGGATTACAAGGATGACGACGATAAGTCGTCCAAGTCGCAATTGAC | pRS415-ScTDH3p-pnpyrG-ScADH1t |
|  | pyrGTAG2 | CAAGATAGTGGCGATAGGGTCAAC |  |
|  | pyrGTAG3 | GATATCATCTCCGATGAAGCCTCCG |  |
|  | tdhTAG4 | CTTATCGTCGTCATCCTTGTAATCcattttgtttgtttatgtgtgtttattcg |  |
| pnPO-FLAG TAG | pyrGTAG1 | ATGGATTACAAGGATGACGACGATAAGTCGTCCAAGTCGCAATTGAC | pRS415-pnpyrGp-pnpyrG-ScADH1t |
|  | pyrGTAG2 | CAAGATAGTGGCGATAGGGTCAAC |  |
|  | pyrGTAG3 | GATATCATCTCCGATGAAGCCTCCG |  |
|  | pnTAG4 | CTTATCGTCGTCATCCTTGTAATCcatggcggggttggcggggtgaagag |  |
| pnAll-FLAG TAG | pyrGtag1 | ATGGATTACAAGGATGACGACGATAAGTCGTCCAAGTCGCAATTGAC | pRS415-pnpyrGp-pnpyrG-pnpyrGt |
|  | pyrGTAG2 | CAAGATAGTGGCGATAGGGTCAAC |  |
|  | pyrGTAG3 | GATATCATCTCCGATGAAGCCTCCG |  |
|  | pnTAG4 | CTTATCGTCGTCATCCTTGTAATCcatggcggggttggcggggtgaagag |  |
| pgP-FLAG TAG | URA3TAG1 | atgGATTACAAGGATGACGACGATAAGtcgaaagctacatataaggaacg | pRS415-PgpyrGp-ScURA3-ScADH1t |
|  | pyrGTAG2 | CAAGATAGTGGCGATAGGGTCAAC |  |
|  | pyrGTAG3 | GATATCATCTCCGATGAAGCCTCCG |  |
|  | pgTAG4 | CTTATCGTCGTCATCCTTGTAATCcatggcggggccgaagagatgtgtatttg |  |
| pgO-FLAG TAG | pyrGTAG1 | ATGGATTACAAGGATGACGACGATAAGTCGTCCAAGTCGCAATTGAC | pRS415-ScTDH3p-PgpyrG-ScADH1t |
|  | pyrGTAG2 | CAAGATAGTGGCGATAGGGTCAAC |  |
|  | pyrGTAG3 | GATATCATCTCCGATGAAGCCTCCG |  |
|  | pgTAG4 | CTTATCGTCGTCATCCTTGTAATCcatggcggggccgaagagatgtgtatttg |  |
| pgPO-FLAG TAG | pyrGTAG1 | ATGGATTACAAGGATGACGACGATAAGTCGTCCAAGTCGCAATTGAC | pRS415-PgpyrGp-PgpyrG-ScADH1t |
|  | pyrGTAG2 | CAAGATAGTGGCGATAGGGTCAAC |  |
|  | pyrGTAG3 | GATATCATCTCCGATGAAGCCTCCG |  |
|  | pgTAG4 | CTTATCGTCGTCATCCTTGTAATCcatggcggggccgaagagatgtgtatttg |  |
| pgAll-FLAG TAG | pyrGTAG1 | ATGGATTACAAGGATGACGACGATAAGTCGTCCAAGTCGCAATTGAC | pRS415-PgpyrGp-PgpyrG-PgpyrGt |
|  | pyrGTAG2 | CAAGATAGTGGCGATAGGGTCAAC |  |
|  | pyrGTAG3 | GATATCATCTCCGATGAAGCCTCCG |  |
|  | pgTAG4 | CTTATCGTCGTCATCCTTGTAATCcatggcggggccgaagagatgtgtatttg |  |
| pdpP-FLAG TAG | URA3TAG1 | atgGATTACAAGGATGACGACGATAAGtcgaaagctacatataaggaacg | pRS415-pdppyrGp-SCura3-ScADH1t |
|  | pyrGTAG2 | CAAGATAGTGGCGATAGGGTCAAC |  |
|  | pyrGTAG3 | GATATCATCTCCGATGAAGCCTCCG |  |
|  | pdpTAG4 | CTTATCGTCGTCATCCTTGTAATCcatggcggggctggcggggtgaagag |  |
| pdpO-FLAG TAG | pyrGTAG1 | ATGGATTACAAGGATGACGACGATAAGTCGTCCAAGTCGCAATTGAC | pRS415-ScTDH3p-pdppyrG-ScADH1t |
|  | pyrGTAG2 | CAAGATAGTGGCGATAGGGTCAAC |  |
|  | pyrGTAG3 | GATATCATCTCCGATGAAGCCTCCG |  |
|  | pdpTAG4 | CTTATCGTCGTCATCCTTGTAATCcatggcggggctggcggggtgaagag |  |
| pdpPO-FLAG TAG | pyrGTAG1 | ATGGATTACAAGGATGACGACGATAAGTCGTCCAAGTCGCAATTGAC | pRS415-pdppyrGp-pdppyrG-ScADH1t |
|  | pyrGTAG2 | CAAGATAGTGGCGATAGGGTCAAC |  |
|  | pyrGTAG3 | GATATCATCTCCGATGAAGCCTCCG |  |
|  | pdpTAG4 | CTTATCGTCGTCATCCTTGTAATCcatggcggggctggcggggtgaagag |  |
| pdpAll-FLAG TAG | pyrGTAG1 | ATGGATTACAAGGATGACGACGATAAGTCGTCCAAGTCGCAATTGAC | pRS415-pdppyrGp-pdppyrG-pdppyrGt |
|  | pyrGTAG2 | CAAGATAGTGGCGATAGGGTCAAC |  |
|  | pyrGTAG3 | GATATCATCTCCGATGAAGCCTCCG |  |
|  | pdpTAG4 | CTTATCGTCGTCATCCTTGTAATCcatggcggggctggcggggtgaagag |  |
| pcsP-FLAG TAG | URA3TAG1 | atgGATTACAAGGATGACGACGATAAGtcgaaagctacatataaggaacg | pRS415-pcspyrGp-ScURA3-ScADH1t |
|  | pyrGTAG2 | CAAGATAGTGGCGATAGGGTCAAC |  |
|  | pyrGTAG3 | GATATCATCTCCGATGAAGCCTCCG |  |
|  | pcsTAG4 | CTTATCGTCGTCATCCTTGTAATCcatggcggggttggcggggtgaagag |  |
| pcsO-FLAG TAG | pcsTAG1 | ATGGATTACAAGGATGACGACGATAAGTCCTCCAAGTCGCAATTGAC | pRS415-ScTDH3p-pcspyrG-ScADH1t |
|  | pyrGTAG2 | CAAGATAGTGGCGATAGGGTCAAC |  |
|  | pyrGTAG3 | GATATCATCTCCGATGAAGCCTCCG |  |
|  | pcsTAG4 | CTTATCGTCGTCATCCTTGTAATCcatggcggggttggcggggtgaagag |  |
| pcsPO-FLAG TAG | pcsTAG1 | ATGGATTACAAGGATGACGACGATAAGTCCTCCAAGTCGCAATTGAC | pRS415-pcspyrGp-pcspyrG -ScADH1t |
|  | pyrGTAG2 | CAAGATAGTGGCGATAGGGTCAAC |  |
|  | pyrGTAG3 | GATATCATCTCCGATGAAGCCTCCG |  |
|  | pcsTAG4 | CTTATCGTCGTCATCCTTGTAATCcatggcggggttggcggggtgaagag |  |
| pcsAll-FLAG TAG | pcsTAG1 | ATGGATTACAAGGATGACGACGATAAGTCCTCCAAGTCGCAATTGAC | pRS415-pcspyrGp-pcspyrG-pcspyrGt |
|  | pyrGTAG2 | CAAGATAGTGGCGATAGGGTCAAC |  |
|  | pyrGTAG3 | GATATCATCTCCGATGAAGCCTCCG |  |
|  | pcsTAG4 | CTTATCGTCGTCATCCTTGTAATCcatggcggggttggcggggtgaagag |  |
| ppaP-FLAG TAG | URA3TAG1 | atgGATTACAAGGATGACGACGATAAGtcgaaagctacatataaggaacg | pRS415-PpapyrGp-ScURA3-ScADH1t |
|  | pyrGTAG2 | CAAGATAGTGGCGATAGGGTCAAC |  |
|  | pyrGTAG3 | GATATCATCTCCGATGAAGCCTCCG |  |
|  | ppaTAG4 | CTTATCGTCGTCATCCTTGTAATCCATggcgTggttggtggagtgggttgat |  |
| ppaO-FLAG TAG | pyrGTAG1 | ATGGATTACAAGGATGACGACGATAAGTCGTCCAAGTCGCAATTGAC | pRS415-ScTDH3p-PpapyrG-ScADH1t |
|  | pyrGTAG2 | CAAGATAGTGGCGATAGGGTCAAC |  |
|  | pyrGTAG3 | GATATCATCTCCGATGAAGCCTCCG |  |
|  | ppaTAG4 | CTTATCGTCGTCATCCTTGTAATCCATggcgGggttggtggagtgggttgat |  |
| ppaPO-FLAG TAG | pyrGTAG1 | ATGGATTACAAGGATGACGACGATAAGTCGTCCAAGTCGCAATTGAC | pRS415-PpapyrGp-PpapyrG-ScADH1t |
|  | pyrGTAG2 | CAAGATAGTGGCGATAGGGTCAAC |  |
|  | pyrGTAG3 | GATATCATCTCCGATGAAGCCTCCG |  |
|  | ppaTAG4 | CTTATCGTCGTCATCCTTGTAATCCATggcgGggttggtggagtgggttgat |  |
| ppaAll-FLAG TAG | pyrGTAG1 | ATGGATTACAAGGATGACGACGATAAGTCGTCCAAGTCGCAATTGAC | pRS415-PpapyrGp-PpapyrG-PpapyrGt |
|  | pyrGTAG2 | CAAGATAGTGGCGATAGGGTCAAC |  |
|  | pyrGTAG3 | GATATCATCTCCGATGAAGCCTCCG |  |
|  | ppaTAG4 | CTTATCGTCGTCATCCTTGTAATCCATggcgGggttggtggagtgggttgat |  |
| pcP-FLAG TAG | URA3TAG1 | atgGATTACAAGGATGACGACGATAAGtcgaaagctacatataaggaacg | pRS415-PcpyrGp-ScURA3-ScADH1t |
|  | pyrGTAG2 | CAAGATAGTGGCGATAGGGTCAAC |  |
|  | pyrGTAG3 | GATATCATCTCCGATGAAGCCTCCG |  |
|  | copTAG4 | CTTATCGTCGTCATCCTTGTAATCcatgacgcggtcgggtgaagagacgggtatgtat |  |
| pcO-FLAG TAG | pyrGTAG1 | ATGGATTACAAGGATGACGACGATAAGTCGTCCAAGTCGCAATTGAC | pRS415-ScTDH3p-PcpyrG-ScADH1t |
|  | pyrGTAG2 | CAAGATAGTGGCGATAGGGTCAAC |  |
|  | pyrGTAG3 | GATATCATCTCCGATGAAGCCTCCG |  |
|  | copTAG4 | CTTATCGTCGTCATCCTTGTAATCcatgacgcggtcgggtgaagagacgggtatgtat |  |
| pcPO-FLAG TAG | pyrGTAG1 | ATGGATTACAAGGATGACGACGATAAGTCGTCCAAGTCGCAATTGAC | pRS415-PcpyrGp-PcpyrG-ScADH1t |
|  | pyrGTAG2 | CAAGATAGTGGCGATAGGGTCAAC |  |
|  | pyrGTAG3 | GATATCATCTCCGATGAAGCCTCCG |  |
|  | copTAG4 | CTTATCGTCGTCATCCTTGTAATCcatgacgcggtcgggtgaagagacgggtatgtat |  |
| pcAll-FLAG TAG | pyrGTAG1 | ATGGATTACAAGGATGACGACGATAAGTCGTCCAAGTCGCAATTGAC | pRS415-PcpyrGp-PcpyrG-PcpyrGt |
|  | pyrGTAG2 | CAAGATAGTGGCGATAGGGTCAAC |  |
|  | pyrGTAG3 | GATATCATCTCCGATGAAGCCTCCG |  |
|  | copTAG4 | CTTATCGTCGTCATCCTTGTAATCcatgacgcggtcgggtgaagagacgggtatgtat |  |
| psoP-FLAG TAG | URA3TAG1 | atgGATTACAAGGATGACGACGATAAGtcgaaagctacatataaggaacg | pRS415-psopyrGp-ScURA3-ScADH1t |
|  | pyrGTAG2 | CAAGATAGTGGCGATAGGGTCAAC |  |
|  | pyrGTAG3 | GATATCATCTCCGATGAAGCCTCCG |  |
|  | psoTAG4 | CTTATCGTCGTCATCCTTGTAATCcatggtggggttggcggggtgaagaagtggt |  |
| psoO-FLAG TAG | pyrGtag1 | ATGGATTACAAGGATGACGACGATAAGTCGTCCAAGTCGCAATTGAC | pRS415-ScTDH3p-psopyrG-ScADH1t |
|  | pyrGTAG2 | CAAGATAGTGGCGATAGGGTCAAC |  |
|  | pyrGTAG3 | GATATCATCTCCGATGAAGCCTCCG |  |
|  | tdhTAG4 | CTTATCGTCGTCATCCTTGTAATCcattttgtttgtttatgtgtgtttattcg |  |
| psoPO-FLAG TAG | pyrGtag1 | ATGGATTACAAGGATGACGACGATAAGTCGTCCAAGTCGCAATTGAC | pRS415-psopyrGp-psopyrG-ScADH1t |
|  | pyrGTAG2 | CAAGATAGTGGCGATAGGGTCAAC |  |
|  | pyrGTAG3 | GATATCATCTCCGATGAAGCCTCCG |  |
|  | psoTAG4 | CTTATCGTCGTCATCCTTGTAATCcatggtggggttggcggggtgaagaagtggt |  |
| psoAll-FLAG TAG | pyrGtag1 | ATGGATTACAAGGATGACGACGATAAGTCGTCCAAGTCGCAATTGAC | pRS415-psopyrGp-psopyrG-psopyrGt |
|  | pyrGTAG2 | CAAGATAGTGGCGATAGGGTCAAC |  |
|  | pyrGTAG3 | GATATCATCTCCGATGAAGCCTCCG |  |
|  | psoTAG4 | CTTATCGTCGTCATCCTTGTAATCcatggtggggttggcggggtgaagaagtggt |  |
|  | **Primers for removing the intron on penicillium pyrG gene** | |  |
| proIFpyrG-FLAG TAG | proC1 | AAAGGAGCTCCTGGACCTCGCCGACCGTCTTGGTCCCTACATCGCCGT | pRS415-ScTDH3p-PropyrG-ScADH1t |
|  | pyrGTAG2 | CAAGATAGTGGCGATAGGGTCAAC |  |
|  | pyrGTAG3 | GATATCATCTCCGATGAAGCCTCCG |  |
|  | proC4 | ACGGCGATGTAGGGACCAAGACGGTCGGCGAGGTCCAGGAGCTCCTT |  |
| pnIFpyrG-FLAG TAG | pnC1 | ctcctggacctcgccgaccgccttggcccctacatcgccgtg | pRS415-ScTDH3p-pnpyrG-ScADH1t |
|  | pyrGTAG2 | CAAGATAGTGGCGATAGGGTCAAC |  |
|  | pyrGTAG3 | GATATCATCTCCGATGAAGCCTCCG |  |
|  | pnC4 | cacggcgatgtaggggccaaggcggtcggcgaggtccaggag |  |
| pgIFpyrG-FLAG TAG | pgC1 | aggagctcctggagcttgccgaccgccttggcccctacatcgccgtg | pRS415-ScTDH3p-PgpyrG-ScADH1t |
|  | pyrGTAG2 | CAAGATAGTGGCGATAGGGTCAAC |  |
|  | pyrGTAG3 | GATATCATCTCCGATGAAGCCTCCG |  |
|  | pgC4 | tcacggcgatgtaggggccaaggcggtcggcaagctccaggagctcct |  |
| pdpIFpyrG-FLAG TAG | pdpC1 | ctcctggacctcgccgaccgccttggcccctacatcgccgtg | pRS415-ScTDH3p-pdppyrG-ScADH1t |
|  | pyrGTAG2 | CAAGATAGTGGCGATAGGGTCAAC |  |
|  | pyrGTAG3 | GATATCATCTCCGATGAAGCCTCCG |  |
|  | pdpC4 | cacggcgatgtaggggccaaggcggtcggcgaggtccaggag |  |
| pcsIFpyrG-FLAG TAG | psoC1 | ctcctggacctcgccgaccgccttggcccctacatcgccgtg | pRS415-ScTDH3p-pcspyrG-ScADH1t |
|  | pyrGTAG2 | CAAGATAGTGGCGATAGGGTCAAC |  |
|  | pyrGTAG3 | GATATCATCTCCGATGAAGCCTCCG |  |
|  | psoC4 | cacggcgatgtaggggccaaggcggtcggcgaggtccaggag |  |
| ppaIFpyrG-FLAG TAG | proC1 | AAAGGAGCTCCTGGACCTCGCCGACCGTCTTGGTCCCTACATCGCCGT | pRS415-ScTDH3p-PpapyrG-ScADH1t |
|  | pyrGTAG2 | CAAGATAGTGGCGATAGGGTCAAC |  |
|  | pyrGTAG3 | GATATCATCTCCGATGAAGCCTCCG |  |
|  | proC4 | ACGGCGATGTAGGGACCAAGACGGTCGGCGAGGTCCAGGAGCTCCTT |  |
| pcIFpyrG-FLAG TAG | psoC1 | ctcctggacctcgccgaccgccttggcccctacatcgccgtg | pRS415-ScTDH3p-PcpyrG-ScADH1t |
|  | pyrGTAG2 | CAAGATAGTGGCGATAGGGTCAAC |  |
|  | pyrGTAG3 | GATATCATCTCCGATGAAGCCTCCG |  |
|  | psoC4 | cacggcgatgtaggggccaaggcggtcggcgaggtccaggag |  |
| psoIFpyrG-FLAG TAG | psoC1 | ctcctggacctcgccgaccgccttggcccctacatcgccgtg | pRS415-ScTDH3p-psopyrG-ScADH1t |
|  | pyrGTAG2 | CAAGATAGTGGCGATAGGGTCAAC |  |
|  | pyrGTAG3 | GATATCATCTCCGATGAAGCCTCCG |  |
|  | psoC4 | cacggcgatgtaggggccaaggcggtcggcgaggtccaggag |  |
|  | **Primers for replacing the intron on pyrG gene with the second MATa1 intron from *Saccharomuces cerevisiae*** | |  |
| Split ScURA3- FLAG TAG | MATa1-P1 | GAGAATCAAACTTAAATATATCCTATACTAACAATTTGTAGggcccaggtattgttagc | pRS415-ScTDH3p-ScURA3-ScADH1t |
|  | pyrGTAG2 | CAAGATAGTGGCGATAGGGTCAAC |  |
|  | pyrGTAG3 | GATATCATCTCCGATGAAGCCTCCG |  |
|  | MATa1-P2 | TGTTAGTATAGGATATATTTAAGTTTGATTCTCATATTACATACcaccacaccgtgtgc |  |
| proMpyrG-FLAG TAG | proM1 | TGTAATATGAGAATCAAACTTAAATATATCCTATACTAACAATTTGTAGGTCTTGGTCCCTACATCGCCGT | pRS415-ScTDH3p-PropyrG-ScADH1t |
|  | pyrGTAG2 | CAAGATAGTGGCGATAGGGTCAAC |  |
|  | pyrGTAG3 | GATATCATCTCCGATGAAGCCTCCG |  |
|  | psoM4 | CAAATTGTTAGTATAGGATATATTTAAGTTTGATTCTCATATTACATACggtcggcgaggtccaggag |  |
| pnMpyrG-FLAG TAG | psoM1 | TGTAATATGAGAATCAAACTTAAATATATCCTATACTAACAATTTGTAGgccttggcccctacatcgccgtg | pRS415-ScTDH3p-pnpyrG-ScADH1t |
|  | pyrGTAG2 | CAAGATAGTGGCGATAGGGTCAAC |  |
|  | pyrGTAG3 | GATATCATCTCCGATGAAGCCTCCG |  |
|  | psoM4 | CAAATTGTTAGTATAGGATATATTTAAGTTTGATTCTCATATTACATACggtcggcgaggtccaggag |  |
| pgMpyrG-FLAG TAG | psoM1 | TGTAATATGAGAATCAAACTTAAATATATCCTATACTAACAATTTGTAGgccttggcccctacatcgccgtg | pRS415-ScTDH3p-PgpyrG-ScADH1t |
|  | pyrGTAG2 | CAAGATAGTGGCGATAGGGTCAAC |  |
|  | pyrGTAG3 | GATATCATCTCCGATGAAGCCTCCG |  |
|  | pgM4 | AAATTGTTAGTATAGGATATATTTAAGTTTGATTCTCATATTACATACggtcggcaagctccaggagctcct |  |
| pdpMpyrG-FLAG TAG | psoM1 | TGTAATATGAGAATCAAACTTAAATATATCCTATACTAACAATTTGTAGgccttggcccctacatcgccgtg | pRS415-ScTDH3p-pdppyrG-ScADH1t |
|  | pyrGTAG2 | CAAGATAGTGGCGATAGGGTCAAC |  |
|  | pyrGTAG3 | GATATCATCTCCGATGAAGCCTCCG |  |
|  | psoM4 | CAAATTGTTAGTATAGGATATATTTAAGTTTGATTCTCATATTACATACggtcggcgaggtccaggag |  |
| pcsMpyrG-FLAG TAG | psoM1 | TGTAATATGAGAATCAAACTTAAATATATCCTATACTAACAATTTGTAGgccttggcccctacatcgccgtg | pRS415-ScTDH3p-pcspyrG-ScADH1t |
|  | pyrGTAG2 | CAAGATAGTGGCGATAGGGTCAAC |  |
|  | pyrGTAG3 | GATATCATCTCCGATGAAGCCTCCG |  |
|  | psoM4 | CAAATTGTTAGTATAGGATATATTTAAGTTTGATTCTCATATTACATACggtcggcgaggtccaggag |  |
| ppaMpyrG-FLAG TAG | psoM1 | TGTAATATGAGAATCAAACTTAAATATATCCTATACTAACAATTTGTAGgccttggcccctacatcgccgtg | pRS415-ScTDH3p-PpapyrG-ScADH1t |
|  | pyrGTAG2 | CAAGATAGTGGCGATAGGGTCAAC |  |
|  | pyrGTAG3 | GATATCATCTCCGATGAAGCCTCCG |  |
|  | psoM4 | CAAATTGTTAGTATAGGATATATTTAAGTTTGATTCTCATATTACATACggtcggcgaggtccaggag |  |
| pcMpyrG-FLAG TAG | psoM1 | TGTAATATGAGAATCAAACTTAAATATATCCTATACTAACAATTTGTAGgccttggcccctacatcgccgtg | pRS415-ScTDH3p-PcpyrG-ScADH1t |
|  | pyrGTAG2 | CAAGATAGTGGCGATAGGGTCAAC |  |
|  | pyrGTAG3 | GATATCATCTCCGATGAAGCCTCCG |  |
|  | psoM4 | CAAATTGTTAGTATAGGATATATTTAAGTTTGATTCTCATATTACATACggtcggcgaggtccaggag |  |
| psoMpyrG-FLAG TAG | psoM1 | TGTAATATGAGAATCAAACTTAAATATATCCTATACTAACAATTTGTAGgccttggcccctacatcgccgtg | pRS415-ScTDH3p-psopyrG-ScADH1t |
|  | pyrGTAG2 | CAAGATAGTGGCGATAGGGTCAAC |  |
|  | pyrGTAG3 | GATATCATCTCCGATGAAGCCTCCG |  |
|  | psoM4 | CAAATTGTTAGTATAGGATATATTTAAGTTTGATTCTCATATTACATACggtcggcgaggtccaggag |  |
|  | **Primers for inserting the penicillium pyrG intron into the URA3** | |  |
| ProURA3-FLAG TAG | proI1 | AAACCCAGCCACCACCCCTCCGACGAAACAGCCTACTAACCCATTCACAGggcccaggtattgttagcggtttg | PRS415-ScTDH3p-ura3-ADH1t |
|  | pyrGTAG2 | CAAGATAGTGGCGATAGGGTCAAC |  |
|  | pyrGTAG3 | GATATCATCTCCGATGAAGCCTCCG |  |
|  | proI4 | TGGGTTAGTAGGCTGTTTCGTCGGAGGGGTGGTGGCTGGGTTTACTTACcaccacaccgtgtgcattcgtaatg |  |
| PnURA3-FLAG TAG | pnI1 | ccagctcctcccccacccctccaagggaacaacccactaacccatccacagggcccaggtattgttagcggtttg |  |
|  | pyrGTAG2 | CAAGATAGTGGCGATAGGGTCAAC |  |
|  | pyrGTAG3 | GATATCATCTCCGATGAAGCCTCCG |  |
|  | pnI4 | gttagtgggttgttcccttggaggggtgggggaggagctgggttcactaaccaccacaccgtgtgcattcgtaa |  |
| PgURA3-FLAG TAG | pgI1 | agtgaacgcaacataccccacccctccaaacagcccactaacccatctacagggcccaggtattgttagcggtttg |  |
|  | pyrGTAG2 | CAAGATAGTGGCGATAGGGTCAAC |  |
|  | pyrGTAG3 | GATATCATCTCCGATGAAGCCTCCG |  |
|  | pgI4 | gatgggttagtgggctgtttggaggggtggggtatgttgcgttcacttaccaccacaccgtgtgcattcgtaatg |  |
| PdpURA3-FLAG TAG | pdpI1 | ctctacccctccaaCgaagcaacccgCtaacAcaacccgCtaacacattcacagggcccaggtattgttagcggtt |  |
|  | pyrGTAG2 | CAAGATAGTGGCGATAGGGTCAAC |  |
|  | pyrGTAG3 | GATATCATCTCCGATGAAGCCTCCG |  |
|  | pdpI4 | gttgTgttaGcgggttgcttcGttggaggggtagagtgggctgggttcacttaccaccacaccgtgtgcattcgt |  |
| PcsURA3-FLAG TAG | pcsI1 | tgaacccagctccccccactccaaaggaacaagccactaaccatccacagggcccaggtattgttagcggtt |  |
|  | pyrGTAG2 | CAAGATAGTGGCGATAGGGTCAAC |  |
|  | pyrGTAG3 | GATATCATCTCCGATGAAGCCTCCG |  |
|  | pcsI4 | tggttagtggcttgttcctttggagtggggggagctgggttcacttaccaccacaccgtgtgcattcgtaatg |  |
| PpaURA3-FLAG TAG | ppaI1 | cccagaccccaccacccctccaacgaaccagcctactaacccattcacagggcccaggtattgttagcggtttg |  |
|  | pyrGTAG2 | CAAGATAGTGGCGATAGGGTCAAC |  |
|  | pyrGTAG3 | GATATCATCTCCGATGAAGCCTCCG |  |
|  | ppaI4 | gttagtaggctggttcgttggaggggtggtggggtctgggttcacttaccaccacaccgtgtgcattcgtaa |  |
| PcURA3-FLAG TAG | pcI1 | gtgaacgcaacctaccccacccctctaaacagcccactaacccatccacagggcccaggtattgttagcggtttg |  |
|  | pyrGTAG2 | CAAGATAGTGGCGATAGGGTCAAC |  |
|  | pyrGTAG3 | GATATCATCTCCGATGAAGCCTCCG |  |
|  | pcI4 | gatgggttagtgggctgtttagaggggtggggtaggttgcgttcacttaccaccacaccgtgtgcattcgtaatg |  |
| PsoURA3-FLAG TAG | psoI1 | aacccagcccaccccacccctccaacgaaacagcccactaacccatccccagggcccaggtattgttagcggtttg |  |
|  | pyrGTAG2 | CAAGATAGTGGCGATAGGGTCAAC |  |
|  | pyrGTAG3 | GATATCATCTCCGATGAAGCCTCCG |  |
|  | psoI4 | gggttagtgggctgtttcgttggaggggtggggtgggctgggttcacttaccaccacaccgtgtgcattcgtaatg |  |
|  |  |  |  |
|  | **Primers for RT-PCR** | |  |
|  | RTACT1-F/R | AGAGTTGCCCCAGAAGAACA/GGCTTGGATGGAAACGTAGA |  |
|  | RTUra3-5F/R | CGTGCTGCTACTCATCCTAG/GACCTAATGCTTCAACTAACTCCA |  |
|  | RTpso-5F/R | GCTCAATCGCACCCCAAT/GACGGTCACATTGCTCTTCTT |  |
|  | RTPgr-5F/R | GCTCAATCGCACCCCAAT/AACGGTGACATTGCTCTTCTT |  |
|  | RTPcs-5F/R | CCCAATCGCACCCCAATC/GGAGACAGTAACATTGCTCTTCTT |  |
|  | RTpro-5F/R | CCCAATCGCACCCCAATC/TCAGCGGAGACGGTAACAT |  |
|  | RTpcop-5F/R | AGGTTGCCGAAGAGAAGAAGA/GCGAGGTCCAGGAGTTCC |  |
|  | RTppa-5F/R | GCCCAATCGCACCCTAATC/AGCGGAGACGGTGACATT |  |
|  | RTpdp-5F/R | CCCAATCACACCCCAACC/TCACATCAGCGGAGACAGT |  |
|  | RTpna-5F/R | GTCCAAGTCGCAATTGACCTAC/TCCTCTTCTCCTCGGCTACT |  |
|  | RTURA3-3F/R | GTCAACAGTATAGAACCGTGGATG/CGTTCACCCTCTACCTTAGCA |  |
|  | RTpso-3F/R | GGTGCCGACTTCATTATCTCTG/ CCTGTTGCTGGTACTGCTTAG |  |
|  | RTpgr-3F/R | GAGGACTTCGTCGTCTTCAC/ CCTGCTGCTGGTATTGCTTA |  |
|  | RTpcs-3F/R | GGCGTCAACCTCTCGTCTA/ TCGGCAGCGGCATAGATA |  |
|  | RTpro-3F/R | CGCAGGACGAGGACTTTG/ AGATACCACGACCAGAGATAATGA |  |
|  | RTpcop-3F/R | GGTGCGGACTTCATCATCTC/ CTGCTGCTGGTACTGCTTAG |  |
|  | RTppa-3F/R | GAGGACTTCGTCGTCTTCAC/ GCAGCGGCATAGATACCA |  |
|  | RTpdp-3F/R | GGCGTCAACCTCTCGTCTA/ TTGCTGCTGGTACTGCTTAAC |  |
|  | RTpn-3F/R | GGCGTCAACCTCTCGTCTAA/ GCAGCGGCGTAGATACCA |  |
