# Supplementary material for: Characterization of cross-species transcription and splicing from Penicillium to Saccharomyces cerevisiae
Source: J Ind Microbiol Biotechnol. 2021 Aug 13;48(9-10):kuab054. doi: 10.1093/jimb/kuab054 (PMC8788760; doi:10.1093/jimb/kuab054)
Supplement: kuab054_Supplemental_File [file kuab054_supplemental_file.zip › Supplementary information Table S2.docx]

**Supplementary information**

**Supplementary table S2.** **Genomic sequence of pyrG genes from *Penicillium* species**

Genomic sequences encoding predicted intron-containing genes *pyrG* from *Penicillium* are shown with intron sequences in red text and start/stop codons are bolded.

| Species | NCBI accession NO. | Gene Coding Sequence 5’-3’  (introns in red text) |
| --- | --- | --- |
| *P. roqueforti* | Submission # 2486702 | **ATG**TCGTCCAAGTCGCAATTGACCTATAGCGTCCGCGCCCAATCGCACCCCAATCCCCTCGCACGCAAGCTTTTCCAAGTCGCGGAAGAAAAGAAGAGCAATGTTACCGTCTCCGCTGATGTGACCACAACAAAGGAGCTCCTGGACCTCGCCGACCGTAAGTAAACCCAGCCACCACCCCTCCGACGAAACAGCCTACTAACCCATTCACAGGTCTTGGTCCCTACATCGCCGTCATAAAAACACACATTGACATCCTCTCCGACTTCAGCCAAGCAACAATCGATGGCCTGAATGCCCTAGCCCAAAAACACAACTTCCTCATCTTCGAAGACCGCAAATTCATCGACATCGGCAACACTGTCCAGAAACAGTACCACAACGGCACCCTCCGTATCTCCGAATGGGCCCACATAATCAACTGCTCCATCCTACCCGGCGAGGGCATAGTCGATGCCCTCGCGCAAACTGCCCAGGCTACTGATTTCCCCTACGGCTCTGAGCGCGGCCTCCTCATCCTCGCCGAGATGACCTCGAAGGGATCCCTCGCAACAGGCGCCTACACCTCCGCCTCTGTCGATATCGCGCGCAAGTACCCCAGCTTTGTGCTCGGCTTTGTCTCGACCCGGTCGCTGGGCGAGGTCGAGTCTACGGAGGCGCCCGCGCAGGACGAGGACTTTGTCGTCTTCACCACTGGCGTCAACCTCTCGTCTAAAGGCGATAAGCTTGGCCAGCAGTACCAGACACCGCAATCGGCTATTGGCCGCGGTGCCGACTTCATTATCTCTGGTCGTGGTATCTATGCCGCTGCGGACCCTGTTGAAGCTGTTAAACAGTACCAGCAGCAGGGCTGGGAGGCGTATCTGGCCCGTGTGGGTGCGCAA**TAG** |
| *P. solitum* | Submission # 2486702 | **ATG**TCGTCCAAGTCGCAATTGACCTATAGCGCTCGCGCTCAATCGCACCCCAATCCTCTTGCGCGCAAGCTTTTCCAAGTCGCTGAAGAAAAGAAGAGCAATGTGACCGTCTCCGCTGACGTGACCACGACAAAGGAGCTCCTGGACCTCGCCGACCGTAAGTGAACCCAGCCCACCCCACCCCTCCAACGAAACAGCCCACTAACCCATCCCCAGGCCTTGGCCCCTACATCGCCGTGATCAAAACCCACATCGACATCCTCTCCGACTTCAGCCAAGAAACAATCGACGGCCTGAACGCCCTAGCCCAAAAGCACAACTTCCTCATCTTCGAAGACCGCAAATTCATCGACATCGGCAACACAGTCCAGAAACAGTACCACAACGGCACCCTCCGCATCTCGGAATGGGCCCACATAATCAACTGCTCCGTCCTTCCTGGCGAGGGCATCGTCCAGGCCCTCGCACAAACCGCCCAGGCCACTGACTTCCCCTACGGCTCCGAGCGCGGCCTCCTCATCCTCGCCGAGATGACCTCCAAGGGATCCCTCGCAACAGGCTCCTACACCTCCGCCTCCGTTGACATCGCGCGCAAGTACCCCAGCTTTGTTCTGGGCTTTGTCTCCACCCGCTCGTTGGGTGAGGTCGAGTCTACTGAGGCGCCCGCCCAGGGCGAGGACTTCGTCGTCTTCACCACTGGCGTCAACCTCTCGTCTAAGGGCGATAAGCTCGGTCAGCAGTACCAGACACCGCAGTCGGCTATTGGCCGCGGTGCCGACTTCATTATCTCTGGTCGTGGTATCTATGCCGCTGCGGACCCTGTTGAGGCTGCTAAGCAGTACCAGCAACAGGGATGGGAGGCGTATCTGGCCCGCGTGGGCGCACAA**TAG** |
| *P. polonicum* | Submission # 2486702 | **ATG**TCGTCCAAGTCGCAATTGACCTATGGCGCCCGCGCTCAATCGCACCCCAATCCTCTTGCGCGCAAGCTTTTCCAAGTCGCCGAAGAAAAGAAGAGCAATGTCACTGTCTCCGCTGACGTGACCACGACAAAGGAGCTCCTGGACCTCGCCGACCGTAAGTGAACCTAGCCCACCCCCACCCCTCCAACGAAACAGCCCACTAACCCATCCCCAGGCCTTGGTCCCTACATCGCCGTGATCAAAACCCACATCGACATCCTCTCCGACTTCAGCCAAGAAACAATCGACGGCCTGAACGCCCTAGCCCAAAAGCACAACTTCCTCATCTTCGAAGACCGTAAATTCATCGACATCGGCAACACAGTCCAGAAACAGTACCACAACGGCACCCTCCGCATCTCCGAATGGGCCCACATAATCAACTGCTCCGTCCTTCCCGGCGAGGGCATCGTCGAGGCCCTCGCACAAACCGCCCAGGCCACTGACTTCCCCTACGGCTCCGAGCGCGGCCTCCTCATCCTCGCCGAGATGACCTCCAAGGGATCCCTCGCAACAGGCTCCTACACCTCCGCCTCCGTCGACATCGCGCGCAAGTACCCCAGCTTTGTTCTGGGCTTTGTCTCCACCCGCTCGCTGGGCGAGGTCGAGTCTACCGAAGCGCCCGCCCAGGGCGAGGACTTCGTTGTATTCACCACTGGCGTCAACCTCTCGTCTAAGGGCGATAAGCTCGGTCAGCAGTACCAGACGCCGCAGTCGGCTATTGGCCGCGGTGCGGACTTCATTATCTCTGGTCGTGGTATCTATGCGGCTGCGGACCCTGTTGAGGCTGCTAAGCAGTACCAGCAGCAGGGATGGGAGGCGTATCTGGCCCGCGTGGGCGCGCAA**TAG** |
| *P. italicum* | Submission # 2486702 | **ATG**TCGTCCAAGTCACAATTGACCTACAGCGCTCGCGCCCAATCACACCCCAATCCCCTCGCGCGCAAGCTTTTCCAAGTCGCCGAAGAGAAGAAGAGCAATGTCACCGTTTCCGCTGACGTGACCACAACAAAGGAGCTCCTGGATCTCGCCGACCGTAAGTGAACCCAGCCCACCCCTACCCCTCCAACGAAACAACCAACTAACACATTCACAGGCCTTGGCCCTTACATCGCCGTAATCAAAACACACATCGACATCCTCTCCGACTTCAGCCAAGAAACAATCGACGGCCTGAACGCCCTAGCCCAAAAGCACAACTTCCTCATCTTTGAAGACCGCAAATTCATCGACATCGGCAACACTGTCCAGAAACAGTACCACAATGGCACCCTCCGCATCTCCGAATGGGCCCACATAATCAACTGCTCCGTCCTACCCGGCGAGGGCATCGTCGAGGCCCTGGCGCAAACCGCCCAGGCCACTGACTTCCCTTACGGCTCTGAGCGCGGCCTCCTCATCCTCGCCGAGATGACCTCCAAGGGATCCCTCGCAACCGGCGCCTACACCTCCGCCTCGGTCGATATCGCGCGCAAGTACCCCAGCTTTGTTCTGGGCTTTGTGTCGACCCGCTCGCTGGGCGAGGTCGAGTCTACCGAGGCTCCTACCCAGGGCGAGGACTTCGTCGTCTTCACTACTGGCGTTAACCTCTCGTCCAAGGGCGATAAGCTCGGTCAGCAATACCAGACACCGCAGTCGGCTATCGGTCGCGGTGCCGACTTTATTATCTCTGGTCGTGGTATCTATGCCGCTGCCGACCCCGTTGAGGCTGCTAAGCAGTACCAGCAGCAGGGCTGGGAGGCATATCTGGCCCGCGTGGGCGCCCAA**TAG** |
| *P. digitatum* | CP060774.1 | **ATG**TCGTCCAAGTCGCAATTGACCTACAGCGCTCGCGCCCAATCACACCCCAACCCCCTCGCACGCAGGCTTTTCCATGTCGCCGAAGAGAAGAAGAGCAACGTCACTGTCTCCGCTGATGTGACCACAACGAAGGAGCTCCTGGACCTCGCCGACCGTAAGTGAACCCAGCCCACTCTACCCCTCCAACGAAGCAACCCGCTAACACAACCCGCTAACACATTCACAGGCCTTGGCCCCTACATCGCCGTGATCAAAACACACATCGACATCCTCTCCGACTTCAGCCAAGCGACAATCGACGGCCTGAATGCCCTGGCCCAGAAGCACAACTTCCTCATCTTCGAAGACCGCAAATTCATCGACATCGGCAACACAGTCCAGAAACAGTACCACAACGGCACCCTCCGCATCTCCGAATGGGCCCATATAATTAACTGCTCTGTCCTGCCGGGTGAGGGCATTGTCGAGGCCCTCGCCCAAACCGCCCAGGCCATTGACTTCCCCTACGGCTCCGAACGCGGCCTCCTCATCCTCGCTGAGATGACTTCCAAGGGATCCCTCGCAACGGGCGCCTATACCTCCGCCTCCGTTGACATCGCGCGTAAGTACCCCAGCTTTGTACTAGGCTTTGTCTCGACCCGCTCGCTGGGTGAGGTCGAGTCAACGGAGGCGCCCGCGCAGGGTGAGGACTTCGTTGTCTTCACCACTGGCGTCAACCTCTCGTCTAAGGGTGATAAGCTTGGCCAGCAGTACCAGACTCCGCAGTCAGCGATTGGCCGCGGTGCCGACTTCATTATCTCGGGTCGTGGTATCTATGCCGCTGCGGACCCTGTTGAGGCCGTTAAGCAGTACCAGCAGCAAGGCTGGGAGGCGTATCTGGCCCGCGTGGGCGCACAA**TAG** |
| *P. paneum* | Submission # 2486702 | **ATG**TCGTCCAAGTCGCAATTGACGTACAGCGTGCGCGCCCAATCGCACCCTAATCCCCTCGCGCGCAAGCTTTTCCAAGTCGCCGAAGAAAAGAAGAGCAATGTCACCGTCTCCGCTGATGTGACCACAACAAAGGAGCTCCTGGACCTCGCCGACCGTAAGTGAACCCAGACCCCACCACCCCTCCAACGAACCAGCCTACTAACCCATTCACAGGTCTTGGTCCCTACATCGCCGTGATAAAGACACACATTGACATCCTCTCCGACTTCAGCCAAGCAACAATCGACGGCTTGAACGCCCTAGCCCAAAAGCACAACTTCCTCATCTTCGAAGACCGCAAATTCATCGACATCGGCAACACTGTCCAGAAACAGTACCACAACGGCACCCTCCGTATCTCCGAATGGGCCCACATAATCAACTGCTCCATCCTACCCGGCGAGGGCATAGTCGACGCCCTCGCGCAAACCGCCCAGGCTCCTGACTTCCCCTACGGCTCCGAGCGCGGCCTCCTCATTCTCGCTGAGATGACCTCGAAGGGATCCCTCGCAACAGGCGCCTACACCTCCGCCTCTGTCGATATCGCGCGCAAGTTCCCCAGTTTCGTGCTGGGCTTTGTCTCGACCCGGTCGCTGGGCGAGGTCGAGTCCACCGAGGCGCCGGCGCAGGACGAGGACTTCGTCGTCTTCACCACTGGCGTTAACCTTTCGTCTAAGGGTGATAAGCTCGGCCAGCAGTACCAGACACCGCAGTCGGCCATTGGCCGTGGTGCCGACTTTATCATCTCTGGTCGTGGTATCTATGCCGCTGCGGACCCTGTTGAAGCTGTTAAGCAGTACCAGCAGCAGGGCTGGGAGGCGTATCTGGCCCGTGTGGGTGCGCAA**TAG** |
| *P. griseofulvum* | Submission # 2486702 | **ATG**TCGTCCAAGTCGCAATTGACCTACAGCGCCCGCGCTCAATCGCACCCCAATCCCCTCGCGCGCAAACTTTTCCAAGTCGCCGAAGAGAAGAAGAGCAATGTCACCGTTTCCGCTGACGTGACCACGACAAAGGAGCTCCTGGAGCTTGCCGACCGTAAGTGAACGCAACATACCCCACCCCTCCAAACAGCCCACTAACCCATCTACAGGCCTTGGCCCCTACATCGCCGTGATCAAAACACACATCGACATCCTCTCCGACTTCAGCCAAGCAACAATTGATGGCCTGAATGCCCTAGCCCAAAAGCACAACTTCCTCATCTTCGAAGACCGCAAATTCATCGACATCGGCAACACAGTCCAGAAACAATACCACAATGGCACCCTCCGCATCTCCGAATGGGCCCACATAATCAACTGCTCCGTCCTACCCGGCGAGGGTATCGTTGAGGCCCTTGCGCAAACTGCCCAAGCCACTGACTTCCCCTATGGCTCCGAGCGCGGCCTCCTCATCCTCGCCGAGATGACCTCGAAGGGATCCCTCGCAACAGGCGCATACACCTCTGCCTCTGTTGATATCGCCCGTAAGTACCCCAGCTTCGTCCTAGGTTTTGTCTCGACCCGGTCGCTGGGCGAAGTCGAGTCTATCGAGGCACCCGCACAGGGCGAGGACTTCGTCGTCTTCACCACCGGCGTCAACCTCTCTTCTAAGGGCGATAAGCTCGGTCAGCAGTACCAGACACCCCAGTCGGCTATTGGCCGTGGTGCGGACTTTATTATCTCTGGTCGTGGTATTTATGCCGCTGCGGACCCTGTTGAGGCCGTTAAGCAATACCAGCAGCAGGGCTGGGAGGCGTATCTGGCCCGCGTGGGTGCGCAA**TAG** |
| *P. rubens* | AM920428.1 | **ATG**GATTACAAGGATGACGACGATAAGTCCTCCAAGTCGCAATTGACCTACAGCGCCCGCGCCCAATCGCACCCCAATCCCCTCGCGCGCAAGCTATTCCAAGTCGCCGAAGAGAAGAAGAGCAATGTTACTGTCTCCGCTGACGTGACCACAACAAAGGAGCTCCTGGACCTCGCCGACCGTAAGTGAACCCAGCTCCCCCCACTCCAAAGGAACAAGCCACTAACCATCCACAGGCCTTGGCCCCTACATCGCCGTGATTAAAACACACATCGACATCCTCTCCGACTTCAGCCAAGAAACAATCGATGGCCTGAACGCCCTAGCGCAAAAGCACAACTTCCTTATCTTCGAAGACCGCAAATTCATCGACATCGGCAACACAGTCCAGAAACAGTACCACAATGGCACCCTCCGCATCTCCGAATGGGCGCACATAATCAACTGCTCCATCCTACCCGGCGAGGGCATTGTCGAGGCCCTCGCTCAAACCGCCCAGGCCACTGATTTCCCCTACGGCTCCGAGCGTGGCCTCCTCATCCTCGCCGAGATGACCTCGAAGGGATCCCTCGCAACAGGCGCCTACACCTCCGCCTCCGTCGACATCGCGCGCAAGTACCCCAGCTTCGTGCTTGGCTTTGTCTCGACCCGGTCTCTCGGCGAGGTCGAGTCTACAGAGGCGCCCGCGCAGGGCGAGGATTTCGTCGTCTTCACCACTGGCGTCAACCTCTCGTCTAAGGGCGATAAGCTCGGTCAGCAGTACCAGACGCCGCAGTCGGCTGTTGGCCGCGGTGCTGACTTTATTATCTCTGGTCGTGGTATCTATGCCGCTGCCGACCCTGTTGAGGCCGCTAAGCAGTACCAGCAGCAGGGCTGGGAGGCGTATCTGGCCCGCGTGGGTGCGCAA**TAG** |
| *P. brasilianum* | Submission # 2486702 | **ATG**TCGTCCAAGTCCCAATTGACCTATGCCGTTCGCGCCGAGAGCCACCCCAACCCTCTGGCTCGCCGACTCTTCCAAGTTGCCGAAGCCAAGAAGAGCAACGTCACCGTCTCCGCCGATGTGACCACCACCAAGGAGCTCCTGGACCTTGCCGACCGTACGTTATCACAAAGTATCACCGTCCAAATCCACACTAACACCCCTCACTTACAGGCCTGGGCCCCTATATTGCCGTGATCAAAACCCACATCGACATCCTCTCCGACTTTAGCGAAGAAACCATTACCGGCCTCAAAGCCCTCGCCACCAAACACAACTTCCTCATCTTCGAAGACCGCAAATTCATCGACATCGGCAACACAGTCCAAAAGCAATACCACAACGGCACCCTCCGCATCTCTGAATGGTCCCACATCATCAACTGCTCCGTCCTGCCCGGTGAAGGCATCGTCGAAGCCCTCGCCCAAACCGCCCAGTCCCCGGACTTCCCTTACGGAAGCGACCGCGGCCTCCTCATCCTCGCCGAGATGACCTCCAAGGGCTCCCTCGCCACCGGTGCGTATACCTCCGCCTCAGTGGACTATGCGCGCAAGTACCACAGCTTTGTGCTGGGCTTTGTGTCGACGAGGGCGCTCGGTGAGGTGGAGAGCAGTGTTGCGCCGGCGGAGGGTGAGGACTTTGTTGTTTTCACGACGGGTGTGAATCTTTCGTCCAAGGGCGATAAGCTGGGCCAGCAGTACCAGACGCCGCAGTCGGCTATCGGTCGTGGTGCGGACTTTATTATCGCTGGTCGGGGTATCTATGCTGCGCCCGATCCCGTTGAGGCTGCCAAGCAGTATCAGCAGCAGGGTTGGGAGGCGTATTTGGCCCGTGTGGGTGGTCAG**TAG** |
| *P. coprophilum* | Submission # 2486702 | **ATG**GATTACAAGGATGACGACGATAAGTCGTCCAAGTCGCAATTGACCTACGGCGCCCGCGCTCAATCGCACCCCAACCCCCTCGCGCGCAAGCTTTTCCAGGTTGCCGAAGAGAAGAAGAGCAATGTCACTGTCTCCGCTGATGTGACCACAACAAAGGAACTCCTGGACCTCGCCGACCGTAAGTGAACGCAACCTACCCCACCCCTCTAAACAGCCCACTAACCCATCCACAGGCCTTGGCCCCTACATCGCCGTGATCAAAACACACATTGACATCCTCTCCGATTTCAGTCAGGAAACAATCGACGGCCTGAACGCTTTGGCCCAGAAGCACAACTTCCTCATCTTCGAGGACCGCAAATTCATCGACATCGGCAACACAGTCCAGAAACAATACCACAACGGCACCCTCCGCATCTCCGAATGGGCCCACATCATCAACTGCTCCGTCCTACCGGGCGAGGGCATTGTCGAGGCCCTCGCGCAAACCGCCCAGTCTACTGACTTCCCTTACGGCTCCGAGCGCGGCCTCCTCATCCTCGCCGAGATGACCTCAAAGGGATCCCTTGCAACGGGCACCTACACCTCCGCCTCCGTCGACATCGCGCGCAAGTACCCCAGCTTTGTGCTGGGCTTTGTCTCAACCCGATCGCTGGGCGAAGTCGAGTCTACCGAGGCCCCCGCACAGGGCGAGGACTTTGTCGTCTTCACCACTGGTGTCAACCTCTCGTCTAAGGGCGATAAGCTCGGTCAGCAGTACCAGACACCCCAGTCAGCTATTGGCCGTGGTGCGGACTTCATCATCTCTGGCCGTGGTATCTATGCCGCTGCAGACCCTGTTGAGGCTGCTAAGCAGTACCAGCAGCAGGGCTGGGATGCGTATCTGGCCCGCGTGGGCGCACAA**TAG** |
| *P. vulpinum* | Submission # 2486702 | **ATG**TCGTCCAAGTCGCAATTGACCTACAGCGCTCGCGCTCAATCGCACCCCAATCCCCTCGCACGCAAGCTTTTCCAAGTCGCCGAAGAGAAGAAGAGCAACGTTACCGTCTCTGCTGACGTGACCACAACAAAGGAGCTCCTGGACCTCGCCGACCGTAAGTGAACTCAGCCTACCCCACCACCCCTCCAACGAAACAACCCTCTAACCTATACACAGGCCTTGGCCCTTACATCGCCGTGATCAAAACACACATCGATATCCTCTCCGACTTCAGCCAAGCAACAATCGACGGCCTGAACGCCCTCGCTCAAAAGCACAACTTCCTCATCTTCGAAGACCGCAAATTCATCGACATCGGCAACACAGTCCAGAAACAATACCACAACGGCACCCTCCGCATCTCCGAATGGGCCCATATTATCAACTGCTCCGTCCTACCCGGCGAGGGCATCGTTGAGGCCCTCGCGCAAACCGCCCAGGCCACTGACTTCCCCTACGGCTCCGAGCGCGGCCTCCTCATCCTCGCCGAGATGACCTCAAAGGGATCTCTCGCAACCGGCGCCTACACCTCCGCTTCCGTCGACATCGCGCGCAAGTACCCTAGCTTTGTGCTGGGCTTTGTCTCGACCCGCTCCCTGGGCGAGGTTGAATCTACTGAGGCCCCCACACAGGGCGAGGACTTCGTCGTCTTCACCACTGGCGTCAACCTCTCTTCTAAGGGCGATAAGCTCGGCCAGCAGTACCAGACGCCACAGTCGGCTATTGGCCGCGGTGCGGACTTTATTATCTCTGGCCGTGGTATCTATGCCGCTGCGGACCCTGTTGAGGCCGCCAAGCAGTACCAGCAGCAGGGCTGGGAGGCGTATCTGGCCCGCGTGGGCGCACAA**TAG** |
| *P. expansum* | Submission # 2486702 | **ATG**TCTTCCAAGTCGCAATTGACCTACAGCGCTCGCGCTCAATCGCACCCCAATCCCCTCGCGCGCAAGCTTTTCCAAGTTGCCGAAGAGAAGAAGAGCAATGTCACCGTCTCCGCTGACGTGACCACAACAAAGGAGCTCCTGGACCTCGCCGACCGTAAGTGAACCCATCCCGCCCTACCCCTCCAACGAAACAACCCACTCACATATTCACAGGCCTTGGCCCTTACATTGCCGTGATCAAAACCCACATCGACATCCTCTCCGACTTCAGCCAAGAAACAATCGACGGCCTGAACGCCCTAGCCCAAAAGCACAACTTCCTCATCTTCGAAGACCGCAAATTCATCGACATCGGCAACACAGTCCAGAAACAGTACCACAACGGCACCCTCCGCATCTCCGAATGGGCCCACATAATCAACTGCTCCGTCCTACCCGGCGAGGGCATCGTCGAGGCCCTCGCGCAAACCGCCCAGGCCACTGACTTCCCCTACGGCTCCGAGCGCGGCCTCCTCATCCTCGCCGAGATGACCTCCAAGGGATCCCTCGCAACGGGCGCCTACACCTCCGCCTCCGTCGACATCGCACGCAAATACCCCAGCTTCGTTCTGGGCTTTGTCTCGACCCGCTCGCTGGGCGAGGTCGAGTCTACCGAGGACCCCACCCAGGGTGAGGACTTTGTCGTCTTCACCACTGGCGTCAACCTCTCGTCCAAGGGTGATAAGCTCGGTCAGCAGTACCAGACGCCGCAGTCGGCTATTGGCCGTGGTGCGGACTTTATTATCTCTGGTCGTGGTATCTACGCCGCTGCGGACCCTGTTGAGGCTGCTAAGCAGTACCAGCAGCAGGGCTGGGAGGCATATCTGGCCCGCGTGGGCGCGCAA**TAG** |
| *P. decumbens* | Submission # 2486702 | **ATG**TCGTCCAAGTCCCAATTGACATACGGCGCTCGCGCCGCCAACCACCCCAACCCGCTGGCTCGGAGGCTCTTCCTGGTCGCCGAGGCCAAGAAGAGCAACGTGACCGTCTCCGCCGACGTCACCACCACAGCCGAACTCCTCGACCTGGCCGACCGTATTATCCTCGCTACCCACACAGAAGACAGAGATACTAACCCTCCGCAGGCCTCGGACCCTACATCGCCGTGATAAAAACCCACATCGACATCCTCTCCGACTTCAGCCAAGAAACAATCAGCGGCCTCAACGCCCTCGCCTCCAAGCACAACTTCCTAATCTTCGAAGACCGCAAATTCATCGACATCGGCAACACCGTTCAAAAGCAGTACCACCAAGGCACCCTCCGCATCTCCGAATGGGCCCACATCATCAACTGCAGCGTACTCCCCGGCGAAGGCATCGTCGAAGCCCTAGCCCAAACCGCCCAGGCCTCTGACTTCCCCTATGGCTCAGACCGCGGCCTTCTCATCCTAGCCGAGATGACCTCCAAGGGCTCCCTAGCAACTGGATCCTACACCTCCGCGTCCGTGGATTATGCGCGCAAATACCAGAGTTTCGTGCTGGGTTTCGTCTCGACCAGATCGTTGAGCGAGGTCGAGTCGACCGTTGCTGCCAAAGAGGATGAGGACTTTGTTGTGTTTACTACTGGCGTTAACCTCTCGTCTAAGGGTGATAAGCTCGGTCAGCAGTATCAGACTCCGCAGTCGGCTATTGGTCGTGGTGCGGATTTCATTATTGCTGGTCGGGGTATCTATAAGGCGGAGGATCCGGTTGAGGCGGCTAAGCAGTATCAGCAGCAGGGGTGGGAGGCGTATTTGGCGCGTGTTGGTGGCCAG**TAG** |
| *P. nalgiovense* | Submission # 2486702 | **ATG**TCGTCCAAGTCGCAATTGACCTACAGCGCCCGCGCCCAATCGCACCCCAATCCCCTCGCGCGCAAGCTTTTCCAAGTAGCCGAGGAGAAGAGGAGCAATGTCACTGTCTCCGCTGACGTGACCACAACAAAGGAGCTCCTGGACCTCGCCGACCGTTAGTGAACCCAGCTCCTCCCCCACCCCTCCAAGGGAACAACCCACTAACCCATCCACAGGCCTTGGCCCCTACATCGCCGTGATTAAAACACACATTGACATCCTCTCCGACTTCAGCCAAGAAACAATCGATGGCCTGAACGCCCTAGCCCAAAAGCACAACTTCCTCATCTTCGAAGACCGCAAATTCATCGACATCGGCAACACAGTCCAGAAACAGTACCACAACGGCACCCTCCGCATCTCCGAATGGGCCCATATAATTAACTGCTCCATCCTACCCGGTGAGGGCATCGTCGAGGCCCTCGCGCAAACCGCCCAGGCTACTGACTTCCCCTACGGCTCCGAGCGCGGTCTCCTCATCCTCGCCGAGATGACCTCGAAGGGATCCCTCGCAACAGGCGCCTACACCTCCGCCTCCGTCGACATCGCGCGCAAGTACCCCAGTTTCGTGCTGGGCTTTGTCTCGACCCGGTCTCTCGGCGAGGTCGAGTCTACAGAGGCGCCCGCGCAGGGCGAGGACTTCGTCGTCTTCACCACTGGCGTCAACCTCTCGTCTAAAGGCGATAAGCTCGGTCAGCAGTACCAGACGCCGCAGTCGGCTGTTGGCCGCGGTGCTGACTTTATTATCTCTGGTCGTGGTATCTACGCCGCTGCCGACCCTGTTGAGGCCGCTAAGCAGTACCAGCAGCAGGGCTGGGAGGCGTATCTGGCCCGCGTGGGTGCGCAA**TAG** |
